# Methyltransferase METTL3 governs the modulation of SH3BGR expression through m6A methylation modification, imparting influence on apoptosis in the context of Down syndrome-associated cardiac development

**DOI:** 10.1038/s41420-024-02164-3

**Published:** 2024-09-06

**Authors:** Weili Shi, Rui Chen, Mingjie Zhou, Yunian Li, Yuwei Zhang, Jikui Wang, Bingtao Hao, Shixiu Liao

**Affiliations:** 1 Henan Provincial People’s Hospital, Medical Genetics Institute of Henan Province, Henan Provincial Key Laboratory of Genetic Diseases and Functional Genomics, People’s Hospital of Zhengzhou University, Zhengzhou, China; 2 National Health Commission Key Laboratory of Birth Defect Prevention, Henan Key Laboratory of Population Defects Prevention, Zhengzhou, China; 3 Henan Provincial People’s Hospital, Department of Obstetrics, People’s Hospital of Zhengzhou University, Zhengzhou, China; 4 Henan Key Laboratory of Medical Tissue Regeneration, School of Basic Medical Sciences, Xinxiang Medical University, Xinxiang, China; 5 Department of Immunology, School of Basic Medical Sciences, Zhengzhou University, Zhengzhou, China

**Keywords:** Gene expression, Mechanisms of disease

## Abstract

Down syndrome (DS), caused by an additional chromosome 21, has a high risk of congenital heart defects (CHD), one of the primary causes of mortality in DS newborns. To elucidate the pathogenetic mechanisms underlying this condition, we explored the role of RNA m6A methylation, regulated by METTL3, in DS cardiac development and its impact on the expression of SH3BGR, a gene located at Down syndrome congenital heart disease (DS-CHD) minimal region. We analyzed DS fetal cardiac tissues to assess RNA m6A methylation levels and identify potential contributors. RNA sequencing was performed to detect differentially expressed genes in the same tissues. To further understand METTL3’s function in heart development, we inactivated *Mettl3* in the developing mouse heart to mimic the significantly reduced METTL3 observed in DS cardiac development. Additionally, human cardiomyocyte AC16 cells were used to investigate the molecular mechanism by which METTL3 regulates SH3BGR expression. Apoptosis was analyzed to evaluate METTL3’s effect on heart development through SH3BGR regulation. Reduced m6A modification and decreased METTL3 expression were observed in human DS fetal hearts, along with a significant increase of SH3BGR expression. METTL3, through m6A modification, was found to regulate SH3BGR expression, by influencing mRNA stability. METTL3-deficient mouse embryos exhibited heart malformation with increased apoptosis, emphasizing its role in heart development. In DS hearts, METTL3 downregulation and SH3BGR upregulation, potentially orchestrated by abnormal m6A modification, contribute to gene dysregulation and apoptosis. This study reveals novel insights into DS cardiac pathology, highlighting the intricate role of METTL3 in DS congenital heart defects and presenting the m6A modification of SH3BGR as a potential therapeutic target.

## Introduction

Down syndrome (DS) is a genetic condition resulting from an additional copy of chromosome 21, leading to a heightened risk of congenital heart defects (CHD). Approximately 40–60% of individuals with DS are affected by CHD [1], serving as one of the primary causes of mortality in infants and children with DS [2]. Notable candidate genes associated with CHD in DS patients, including DSCAM, COL6A2, COL6A1, KCNJ6, and RCAN1, have been identified in multiple studies [3, 4]. The exploration of new candidate genes is crucial for the development of novel diagnostic biomarkers and therapeutic interventions.

The m6A RNA methylation, facilitated by methyltransferases (writers), demethylases (erasers), and m6A binding proteins (readers), represents the most prevalent internal modification of RNA, operating dynamically and reversibly [5]. This modification plays a crucial role in regulating cardiac gene expression and serves as an essential posttranscriptional regulatory mechanism in human heart diseases [6, 7]. Proper m6A deposition, along with the presence of m6A methyltransferase like 3 (METTL3), is indispensable for the differentiation of embryonic stem cells into cardiomyocytes, a process crucial for heart development [8]. In our prior study, we observed a reduction in m6A modification in DS brain tissues, correlated with decreased METTL3 expression [9]. Additionally, we identified the NRIP1 gene, specifically increased in expression in DS brains, as regulated by METTL3, suggesting its potential contribution to DS brain pathology. Despite these observations, the role of m6A modification in DS heart development remains unexplored.

The *SH3BGR* gene, encoding a SH3 domain-binding glutamic acid-rich protein, is located on chromosome 21, within the genetic region associated with Down syndrome. It encodes a protein involved in signal transduction pathways, regulating cell growth, differentiation, and apoptosis. *SH3BGR* demonstrates high expression in the heart and skeletal muscle [10], and its precise level is crucial for heart development [11]. Upregulation of SH3BGR has been reported in failing hearts of human patients with hypertrophic cardiomyopa [12]. Dysregulation of SH3BGR has been associated with altered cell apoptosis and viability in neonatal rat ventricular cardiomyocytes. However, the specific roles of SH3BGR in Down syndrome cardiac development remain unclear. Additionally, it is unknown whether SH3BGR is regulated by m6A modification.

In the present study, we observed a decrease in both m6A modification and the m6A methyltransferase METTL3 in fetal hearts of Down syndrome. Examination of differentially expressed genes revealed a significant increase in the expression of SH3BGR in fetal DS cardiac tissues. *Mettl3* conditional knockout (cKO) mice exhibited heart malformation and lethality before or on the day of birth. We further validated METTL3’s role in regulating the expression of SH3BGR using the human cardiomyocyte AC16 cell line. Subsequent experiments showed that METTL3 regulates SH3BGR expression through m6A modification, impacting *SH3BGR* mRNA stability. In addition, METTL3’s regulation of *SH3BGR* expression was found to influence apoptosis in both cell line and cKO mice. This study sheds light on abnormal m6A modification and emphasizes the essential role of METTL3 in DS heart development. It also unveils a new mechanism underlying the elevated expression of SH3BGR in DS cardiac tissue, providing a potential diagnostic marker and therapeutic target for DS.

## Results

### Decreased m6A modification and the m6A methyltransferase METTL3 were observed in human DS fetal cardiac tissue

**Fig. 1:**
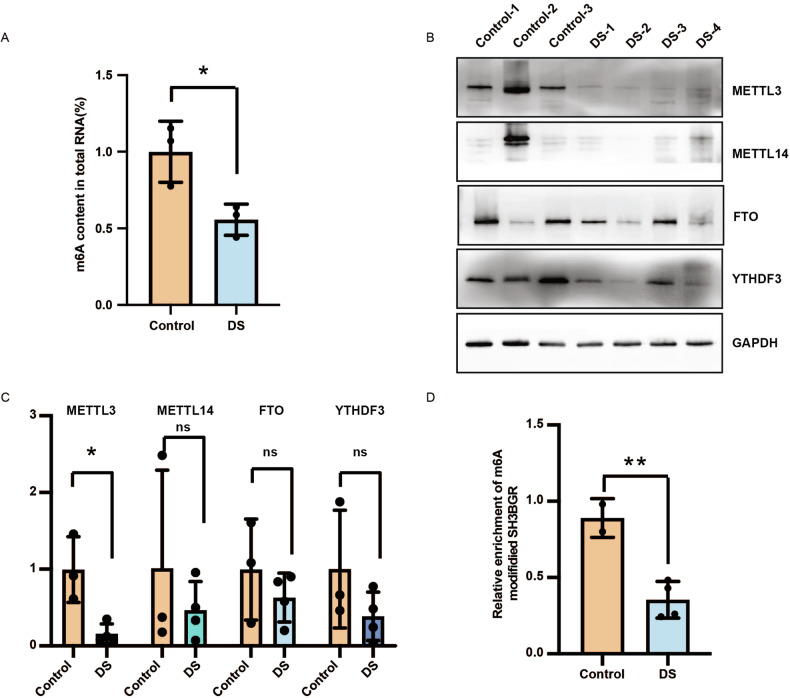
Total RNA m6A modification and METTL3 expression are decreased in DS cardiac tissues. **A** Decreased global m6A level in total RNA isolated from DS fetal cardiac tissue compared with control via an m6A enzyme-linked immunosorbent assay kit. **B** Western blotting analysis of METTL3, METTL14, FTO and YTHDF3 in fetal cardiac tissues of four DSs and three controls. The GAPDH was used as an internal control. **C** Quantification of western blotting for different proteins. **D** The abundance of m6A modified SH3BGR mRNA was reduced in DS compared with control sample. Data were presented as mean ± SD of three independent experiments. *p < 0.05; **p < 0.01 compared with the control group.

Previously, we elucidated that the increased gene expression in DS brain was partially contributed by m6A modification [9]. To explore m6A modification in DS fetal heart, the levels of m6A content in total RNAs in the DS and control samples were measured using enzyme-linked immunosorbent assays. It was observed that the level of m6A modification was significantly decreased in DS cardiac tissues (Fig. 1A). The m6A modification is required for the differentiation of embryonic stem cells into cardiomyocytes, which is critical for normal heart development and function [8]. It plays a crucial role in regulating the expression of developmental regulators via post-transcriptional regulatory mechanisms [13]. As the abnormal m6A modification was involved in human heart disease [6], the significant reduction of m6A modification could be a contributor to DS-CHD. Since m6A modification is mainly catalyzed by the m6A methltransferase complex METTL3/METTL14 and removd by the demethylases FTO and ALKBH5, we speculated that the abnormal m6A modification in DS cardiac tissue was caused by the dysregulation of the methyltransferase and/or demethylases. Then we examined the expression levels of key m6A writers and erasers in DS cardiac tissues. The results showed that METTL3 was significantly reduced in DS, while the expression of METTL14 and FTO in the heart did not change significantly (Fig. 1B–C). SH3BGR was highly expressed in heart, which is essential to heart function. We also tested the m6A modification in the SH3BGR mRNA. The m6A modified SH3BGR mRNA was reduced about two folds in DS samples (Fig. 1D). These data suggested that METTL3 may be largely responsible for the reduced m6A modification in the DS heart.

### Transcriptomic analysis reveals dysregulated pathways in human DS fetal hearts

**Fig. 2:**
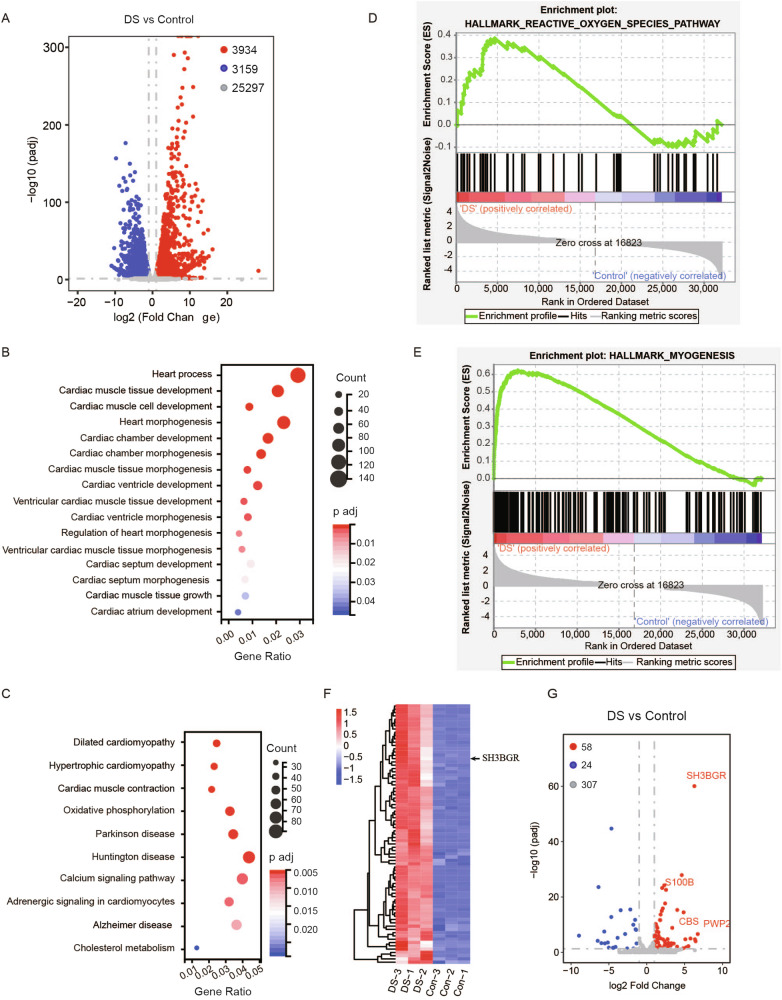
The RNA-seq analysis revealed alterations in the expression profile of DS fetal cardiac tissues. **A** Volcano plot showing log2 fold change of gene expression and corresponding adjust p-value. **B** GO analysis of differentially expressed genes identified by RNA-seq in DS compared with controls. **C** KEGG analysis of differentially expressed genes in DS compared with controls. **D**, **E** GSEA analysis revealed up-regulated pathway in reactive oxygen species and myogenesis. **F** Heatmap for myogenesis. **G** Volcano plot showing transcriptome located on chromosome 21. SH3BGR was one of highly increased genes in DS cardiac tissues.

To explore potential targets of METTL3 in DS heart development, we conducted RNA-Seq of cardiac tissues from DS fetuses. The analysis revealed a 3934 upregulated and 3159 down-regulated genes in DS tissues (Fig. 2A). Gene Ontology (GO) analysis indicated that the differentially expressed genes (DEGs) were associated with cardiac chamber development, cardiac ventricle development, ventricular cardiac muscle tissue morphogenesis (Fig. 2B). KEGG analysis further highlighted the significant involvement of pathways such as cardiac muscle contraction and dilated cardiomyopathy in DS cardiac development (Fig. 2C). To delve deeper into functional differences between DS and control cardiac tissue, Gene Set Enrichment Analysis (GSEA) analysis was carried out with the criteria of p-value < 5% and FDR q-val<25% for significance. Reactive oxygen species were significantly upregulated pathway in the DS group (Fig. 2D), consistent with previous reports [14, 15]. Myogenesis was also observed to be upregulated in DS fetal cardiac tissue (Fig. 2E), suggesting a compensatory response to the disease. Notably, a group of genes, including SH3BGR, were in this gene sets (Fig. 2F). Additionally, an expression analysis of DEGs on chromosome 21 revealed that the majority of genes were upregulated in DS samples (Supplementary Table 1), with SH3BGR being one of the most significantly increased (Fig. 2G).

### SH3BGR was highly upregulated in human DS cardiac tissues

**Fig. 3:**
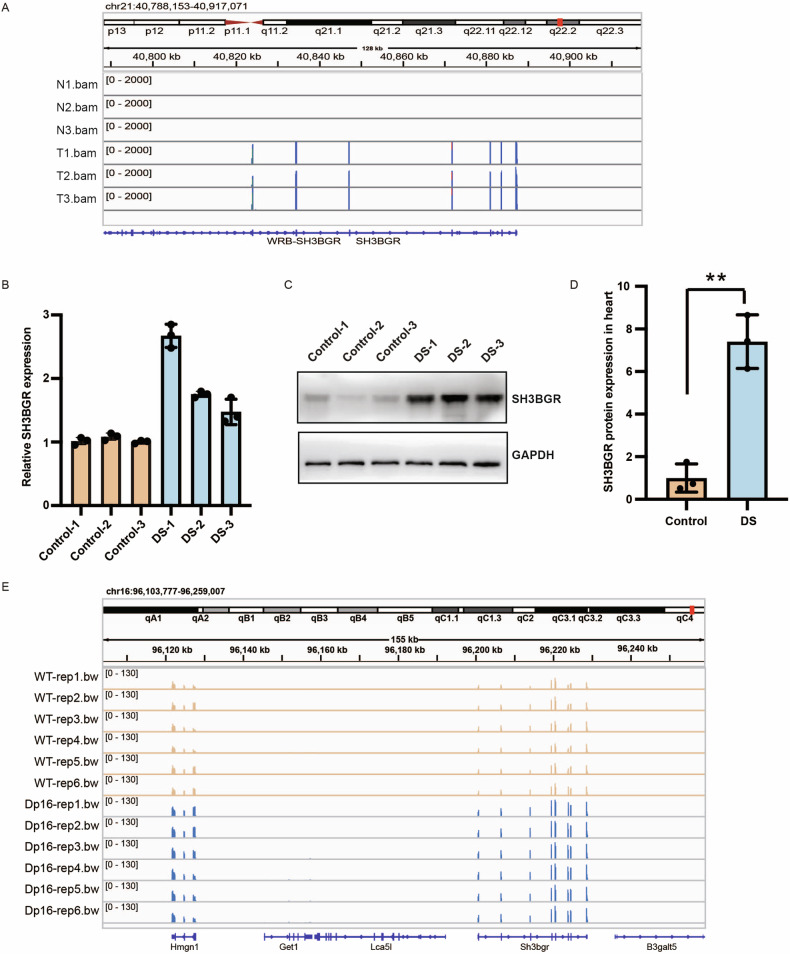
The expression of SH3BGR is dysregulated in DS cardiac tissues. **A** RNA-seq data showed increased expression of SH3BGR in DS cardiac tissues. **B** RT-qPCR analysis confirmed that SH3BGR mRNA was increased in DS tissues. **C**, **D** SH3BGR protein level was analyzed by western blotting in DS and control samples. Data were presented as mean ± SD of three independent experiments. **p < 0.01 compared with the control group. **E** RNA-seq data showed upregulated expression of SH3BGR in heart tissues of Dp16 mice.

The expression of SH3BGR was upregulated in DS cardiac tissue compared to control samples, as revealed by RNA-seq analysis (Fig. 3A). This finding was further confirmed through RT-qPCR analysis in DS cardiac tissues (Fig. 3B). Additionally, the SH3BGR protein levels were elevated in DS, exhibiting an increase of seven folds (Fig. 3C, D). Interestingly, the results indicated that the increase in SH3BGR protein exceeded its RNA level, suggesting a post-transcriptional modification. We then examined the expression of SH3BGR in Dp16 mice, one of the DS mouse models. Sequencing data were downloaded from GEO datasets on the NCBI website (accession number: GSE218888). The expression of SH3BGR was also increased in the heart tissues of Dp16 mice at E12.5 (Fig. 3E). These results suggested that the dysregulation of SH3BGR was highly involved in DS heart development.

### Mettl3 deficiency in mice leads to impaired cardiac development and lethality before birth

**Fig. 4:**
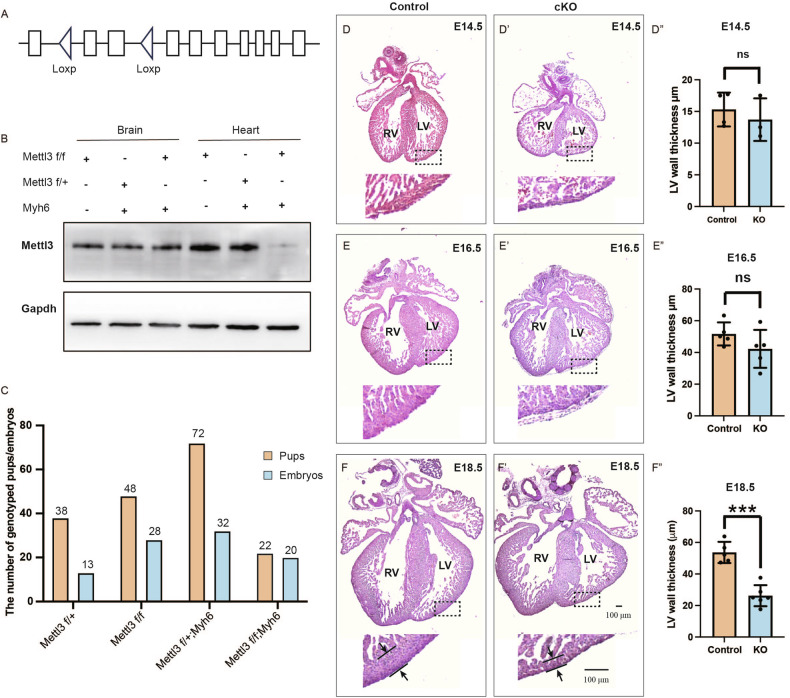
METTL3 deficient mice displayed Cardiac left ventricular malformation. **A** Schematic diagram of generating *Mettl3*^*f/f*^ *mice*. **B** Western blotting analysis confirming the knockout efficiency of Mettl3 in the hearts from cKO mice. Gapdh served as an internal control. **C** Number of mice genotyped in pups and embryos when mating *Mettl3*^*f/f*^ mice with *Myh6-Cre* transgenic mice. Representative HE staining of the hearts from cKO mice and control mice at E14.5 **D**-**D**’, E16.5 **E**-**E**’, E18.5 **F**-**F**’. **D**”, **E**”, **F**” Left wall thickness in cKO and control embryos were measured and calculated respectively.

To investigate the impact of METTL3 inhibition in heart development, we generated a *Mettl3* conditional knockout mice by crossing *Mettl3*^*f/f*^ mice (Fig. 4A) with *Myh6*-*Cre* transgenic mice. The genotype of *Mettl3*^*f/f*^; *Myh6-Cre* was identified as conditional knockout mice (cKO). Western blotting confirmed the absence of METTL3 protein in the cardiac tissue of cKO, while METTL3 protein levels in other tissues, such as the brain, remained unaffected (Fig. 4B). The cKO mice exhibited lethality before or on the day of birth, underscoring the essential role of METTL3 in cardiac development. We mated *Mettl3*^*f/f*^ mice with *Mettl3*^*f/+*^; *Myh6-Cre* mice, genotyping a total of 180 pups and 93 embryos. The number of *Mettl3*^*f/f*^; *Myh6-Cre* in embryos was closely to expected, while it was significantly lower in the pup group (Fig. 4C). As all cKO mice were unable to survive after birth, our focus shifted to cKO embryos to examine the role of METTL3 in cardiac development. Moreover, we analyzed the differentially expressed genes between the RNA-seq dataset from Dp16 embryonic mouse heart tissue and the Mettl3 cKO RNA-seq dataset. Several overlapping genes related to heart development or inflammatory response were identified, as shown in Supplementary Fig. 1. The results suggested that some common expression patterns were shared between these two mouse models, indicating that the cKO mouse could represent aspects of the DS mouse to some extent.

Further histological examination revealed indistinguishable hearts between cKO and controls at E14.5, with mean left ventricular wall thickness of 13.70 ± 3.36 µm (*n* = 3) and 15.30 ± 2.67 µm (*n* = 4), respectively (Fig. 4D, D’, and D”). Similarly, at E16.5, no significant differences were observed, with measurements of 42.26 ± 11.99 µm (*n* = 5) for cKO and 51.70 ± 7.30 µm (*n* = 5) for controls (Fig. 4E, E’, and E”). However, by E18.5, severe thinning of the left ventricular (LV) myocardium and a corresponding decrease in left wall thickness were evident in cKO embryos compared to littermate controls, with mean LV thickness of 26.23 ± 6.64 µm (*n* = 6) and 53.68 ± 6.72 µm (*n* = 5), respectively (Fig. 4F, F’, and F”). In mutants, an enlarged heart (one out of nine embryos) and ventricular septal defect (two out of nine embryos) were also observed (Supplementary Fig. 2). These findings underscore the necessity of METTL3 for proper cardiac left ventricular development.

### The expression of SH3BGR is regulated by the METTL3-mediated m6A modification

**Fig. 5:**
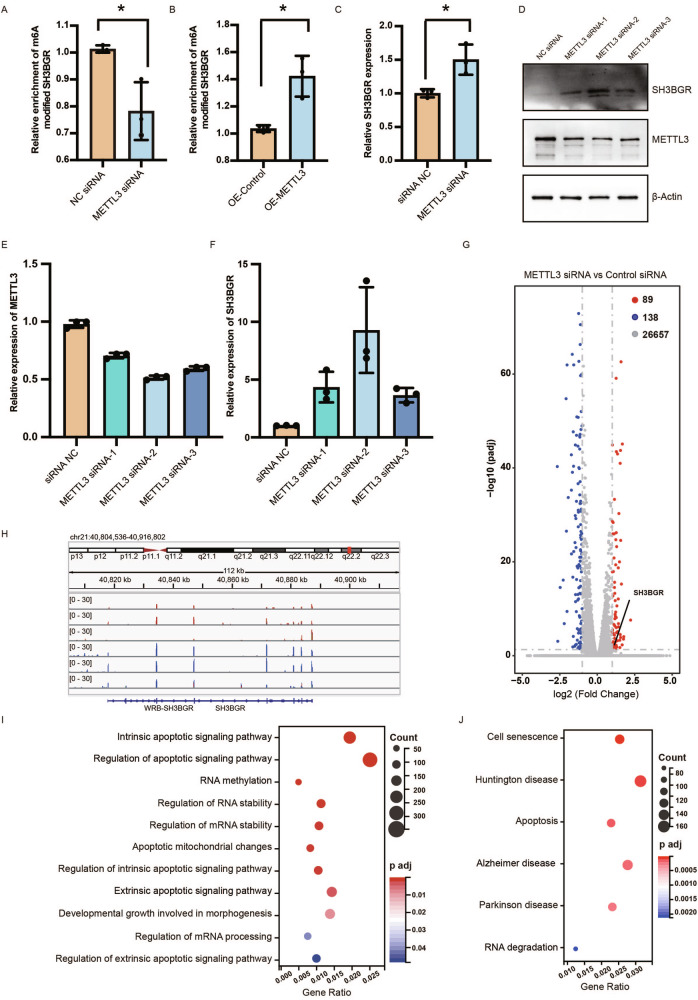
METTL3 regulated the expression of SH3BGR in AC16 cells. **A** Depletion of METTL3 decreased abundance of m6A-modified SH3BGR mRNA in AC16 cells. **B** Overexpression of METTL3 induced SH3BGR mRNA m6A modification in AC16 cells. **C** RT-qPCR analysis of SH3BGR in control and METTL3 knockdown AC16 cells. **D** Western blotting analysis of METTL3 and SH3BGR with or without METTL3 knockdown in AC16 cells. **E**, **F** Statistically analysis of protein expression detected in (**D**). **G** Volcano plot showing log2 fold change of gene expression and corresponding adjust p-value in METTL3 knockdown compared with control AC16 cells. **H** RNA-seq data showed increased expression of SH3BGR in METTL3 knockdown AC16 cells. **I** GO analysis of differentially expressed genes in METTL3 knockdown compared with control AC16 cells. **J** KEGG analysis of differentially expressed genes in METTL3 knockdown compared with control AC16 cells.

To confirm whether SH3BGR is a substrate of METTL3, we performed a MeRIP-qPCR assay to detect m6A-modified SH3BGR mRNA in METTL3-overexpressed or METTL3-knockdown AC16 cells. METTL3 overexpression increased m6A-modified SH3BGR, while METTL3 knockdown reduced m6A-modified SH3BGR mRNA in AC16 cells (Fig. 5A, B). Moreover, SH3BGR mRNA was increased in METTL3 knockdown AC16 cells (Fig. 5C). The protein expression of SH3BGR also was increased when METTL3 was inhibited (Fig. 5D–F). These data suggested that METTL3-mediated m6A modification represses the SH3BGR protein level.

To identify the potential molecular mechanism when METTL3 was inhibited, RNA-seq was performed in AC16 cells. We identified 89 upregulated genes and 138 downregulated genes in METTL3 siRNA transfected cells compared to control siRNA transfected AC16 cells (Fig. 5G, Supplementary Table 2). SH3BGR exhibited a significant increase in METTL3-siRNA-transfected cells ((Fig. 5G–H). GO analysis showed enrichment in RNA methylation, regulation of RNA stability, regulation of apoptotic signaling pathway (Fig. 5I). KEGG analysis revealed enrichment in RNA degradation and apoptosis pathway (Fig. 5J). These results indicated that the reduction of METTL3 enhanced the expression of SH3BGR and suggested the involvement of RNA stability and apoptosis when METTL3 was inhibited in cardiomyocytes.

### METTL3 modulates SH3BGR expression by regulating mRNA stability through m6A modification

**Fig. 6:**
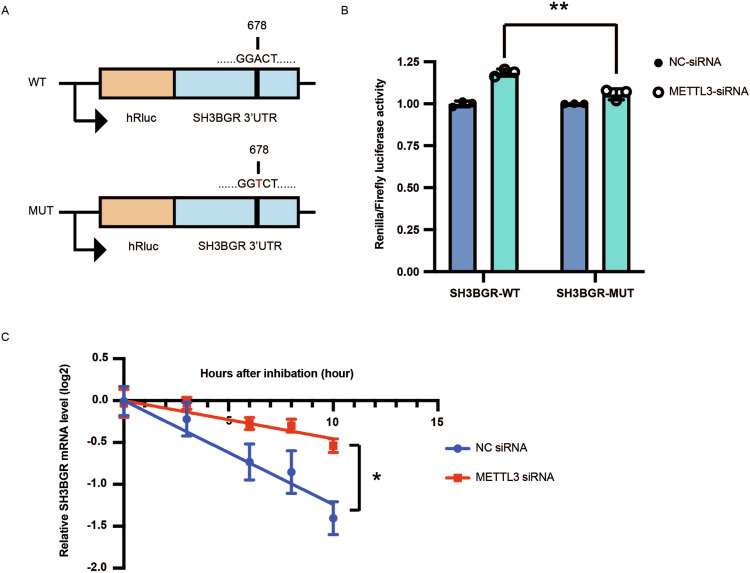
METTL3 regulates the expression of SH3BGR via m6A modification by modulating mRNA stability. **A** A candidate m6A motif on 3′ UTR of SH3BGR was predicted via SRAMP prediction online. Luciferase reporter constructs containing human SH3BGR 3′ UTR that have m6A motif or mutant (A-to-G mutation) m6A site. **B** Relative luciferase activities of AC16 cells cotransfected with plasmids containing wild-type or mutant SH3BGR 3′ UTR and METTL3 siRNA or control siRNA, respectively. **C** Renilla luciferase activities were tested and further normalized to firefly luciferase activity. The decay rate of SH3BGR mRNA was measured in AC16 cells under control siRNA or METTL3 siRNA after actinomycin D treatment and comparison of remaining mRNA by RT-qPCR. Data were presented as mean ± SD of three independent experiments. **P < 0.01, ***P < 0.0001 compared with the control group.

To further investigate whether METTL3 regulate the expression of SH3BGR via m6A modification, we analyzed the sequence of SH3BGR mRNA and identified DRACH motifs (where D = A, G, or U; R = purine; and H = A, C, or U) in the 3′ UTR region using an online m6A modification prediction program (SRAMP) (http://www.cuilab.cn/sramp). Subsequently, the 3′ UTR sequence and an m6A motif-mutated 3′ UTR (where the A in the motifs was mutated to T) of SH3BGR were individually inserted into a luciferase reporter construct (Fig. 6A). Luciferase activity was then measured in transfected AC16 cells. Upon METTL3 knockdown, luciferase activity substantially increased in cells transfected with the reporter containing wild-type 3′ UTR, while this change was abrogated in cells transfected with the mutated 3′ UTR (Fig. 6B). These results indicated that METTL3 regulates the expression of SH3BGR, at least partially in an m6A-dependent manner.

Next, to investigate whether SH3BGR mRNA decay was involved in this process, we measured mRNA levels in AC16 cells treated with the transcription inhibitor actinomycin D during METTL3 knockdown. *SH3BGR* mRNA levels were measured at 0, 3, 6, 8, and 10 h after actinomycin D treatment. METTL3 knockdown significantly increased SH3BGR mRNA stability, with the half-life of SH3BGR mRNA increasing from 4.0 h in control cells to 11.0 h in knockdown cells (Fig. 6C). These results suggested that METTL3-mediated m6A modification promoted the decay of *SH3BGR* mRNA.

### METTL3-mediated regulation of SH3BGR expression plays a crucial role in inhibiting apoptosis

**Fig. 7:**
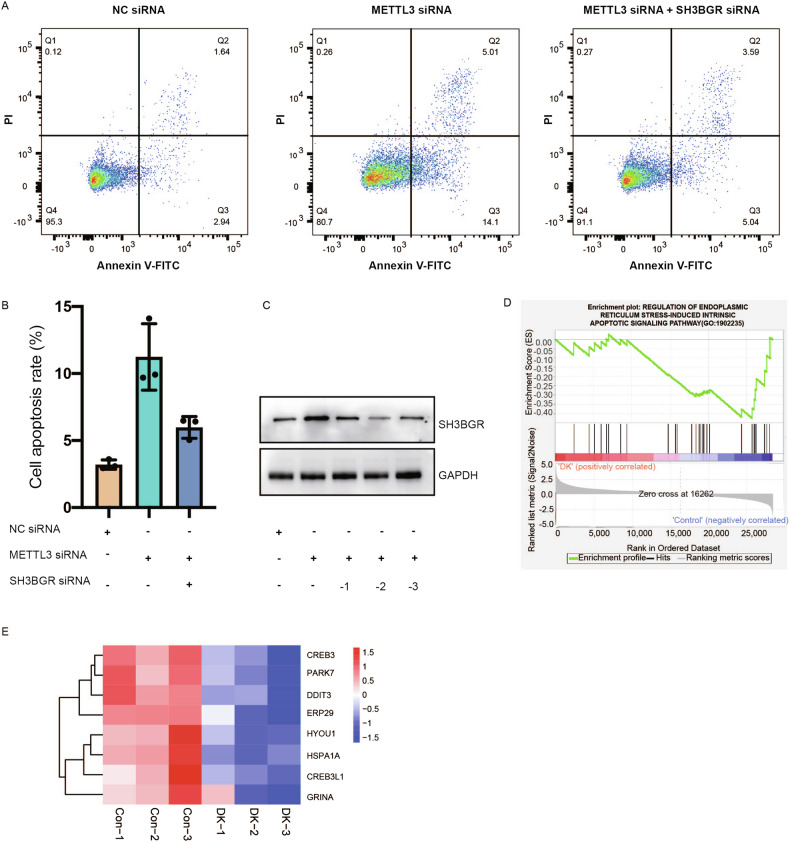
METTL3-mediated regulation of SH3BGR expression influencing apoptosis. **A** Cell apoptosis was examined by flow cytometry in METTL3 knockdown or both METTL3 and SH3BGR knockdown AC16 cells. **B** Statistically analysis of (**A**). **C** Western blotting detected the expression of SH3BGR in METTL3 knockdown or both METTL3 and SH3BGR knockdown AC16 cells. -1, -2, -3 represents three different METTL3 siRNAs. GAPDH served as an internal control. **D** GSEA analysis revealed down-regulated pathway in regulation of intrinsic apoptotic signaling pathway in double knockdown AC16 cells compared to control cells. **E** Heatmap for intrinsic apoptotic signaling pathway.

To investigate the influence of METTL3-mediated regulation of SH3BGR expression on apoptosis in cardiac development, we examined cell apoptosis in METTL3-knockdown AC16 cells. METTL3 knockdown significantly increased early apoptotic cells by around 4-fold, while late apoptotic cells increased by 2-3 times (Fig. 7A and B). To assess the specific role of SH3BGR upregulation in apoptosis caused by METTL3 downregulation, we further knocked down *SH3BGR* in METTL3 knockdown cells, restoring its level to that before METTL3 knockdown (Fig. 7C). Flow cytometric analysis results revealed that SH3BGR knockdown effectively reduced apoptosis caused by METTL3 knockdown, although it did not fully restore it to the level of METTL3 non-knockdown (Fig. 7A and B). These findings demonstrate the crucial role of METTL3 in resisting apoptosis during heart development, with SH3BGR identified as a significant downstream gene contributing to apoptosis in the context of reduced METTL3 expression.

Subsequently, double knockdown AC16 cells were subjected to RNA-Seq to explore potential mechanisms. Apoptosis was not significantly enriched in either GO or KEGG analysis in DK cells. However, GSEA analysis demonstrated a significant reduction in the ‘regulation of intrinsic apoptotic signaling pathway’ in double knockdown AC16 cells compared to control cells (Fig. 7D). A group of genes related to this signaling pathway showed alterations in expression during double knockdown in AC16 cells (Fig. 7E). These findings suggest that METTL3-mediated regulation of SH3BGR expression influences apoptosis in AC16 cells in vitro.

**Fig. 8:**
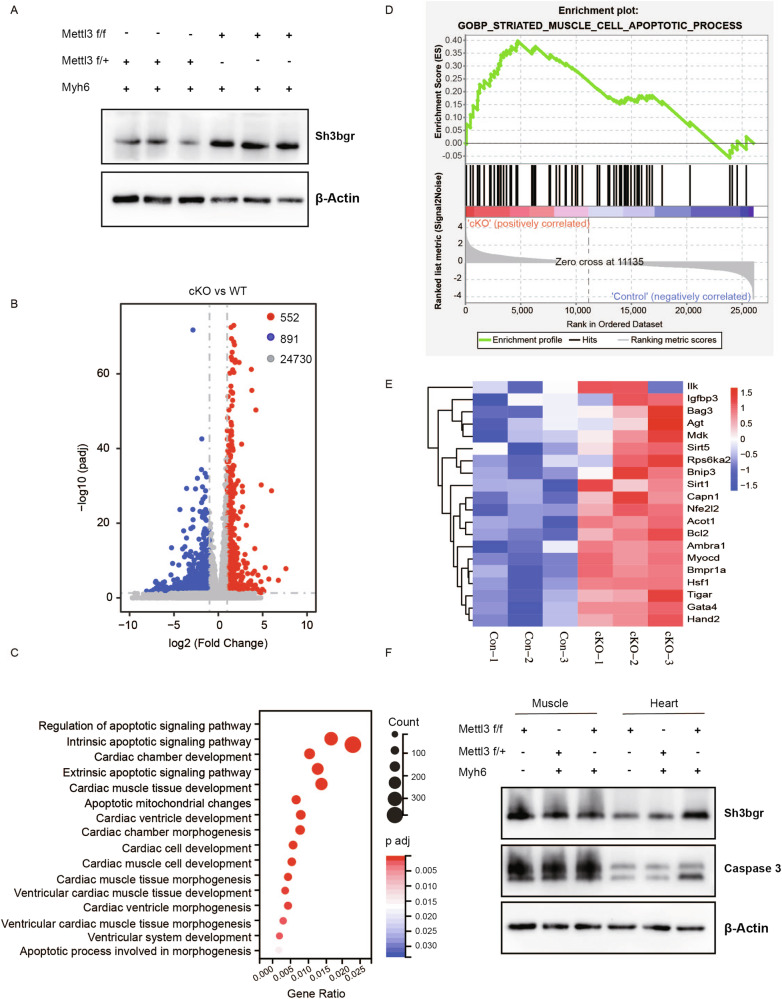
METTL3 targets SH3BGR affecting cell apoptosis in cKO mice. **A** Sh3bgr protein level was analyzed by western blotting in the cardiac tissue of cKO and control mice. Gapdh served as an internal control. **B** Volcano plot showing log2 fold change of gene expression and corresponding adjust p-value in cKO compared with control mice. **C** GO analysis of differentially expressed genes in the cardiac tissue of cKO compared with controls. **D** GSEA analysis revealed up-regulated pathway in striated muscle cell apoptotic process. **E** Heatmap for striated muscle cell apoptotic process. **F** Western blotting analysis examined the expression of Sh3bgr and Caspase 3 in the hearts from cKO and control mice. β-Actin served as an internal control.

To further substantiate the regulatory relationship between METTL3 and SH3BGR in heart development, we assessed the expression of SH3BGR in the hearts of METTL3-deficient mice. The results unequivocally demonstrated a significant upregulation of SH3BGR protein levels in the hearts of METTL3-deficient mice (Fig. 8A), providing compelling evidence that SH3BGR is indeed a direct target of METTL3 in the context of heart development. To unravel the molecular implications of METTL3 deficiency, we performed RNA-Seq on embryos at E18.5, revealing a total of 552 upregulated and 891 downregulated genes in the METTL3-deficient group (Fig. 8B). Strikingly, apoptosis emerged as a prominently enriched pathway in the METTL3-deficient group when analyzing differentially expressed genes (Fig. 8C). Specifically, the striated muscle cell apoptotic process was notably heightened in the METTL3-deficient group (Fig. 8D), accompanied by differential expression of a specific set of genes (Fig. 8E). Since cleaved caspase 3 was considered as a reliable marker for cell apoptosis. Next, we examined the expression of caspase 3 in METTL3-deficient mice. The results showed cleaved Caspase 3 was enhanced in cardiac tissues, while this change was not observed in the muscles (Fig. 8F). These comprehensive findings strongly affirm that METTL3, at least partially through its regulatory influence on SH3BGR expression, plays a pivotal role in modulating apoptosis during heart development in METTL3-deficient embryos.

## Discussion

The proper formation and function of the heart are essential for embryonic survival, necessitating a thorough understanding of the mechanisms governing cardiac development. Post-transcriptional m6A RNA methylation, a vital aspect of RNA functionality, has been implicated in various human heart diseases [16]. Notably, a decreased expression of METTL3 has been observed in failing human hearts [17]. Building on this, our investigation found that METTL3-mediated m6A modification is reduced in DS brains, concurrent with the upregulation of the gene NRIP1 on chromosome 21 [9]. The current study adds a new dimension by demonstrating the downregulation of m6A modification in the DS fetal heart, suggesting that abnormal m6A modification of mRNA may play a pivotal role in the molecular pathology underlying multiple organ abnormalities in DS. However, the specific reason for the downregulation of METTL3 in the brain and heart of individuals with DS remains unclear. Further research is warranted to unravel the intricate factors contributing to this observed downregulation and its implications in DS pathology.

The gene *SH3BGR*, residing in the Down syndrome congenital heart disease (DS-CHD) minimal region, is selectively transcribed in the heart and skeletal muscle [18]. Our investigation revealed a significant upregulation of SH3BGR, particularly at the protein level, in DS fetal cardiac tissue. Given the known regulatory role of m6A modification in the 3′ UTR of mRNA [19], our findings indicate that METTL3 methylates a site in the 3′ UTR of SH3BGR mRNA, thereby regulating its expression. This establishes SH3BGR mRNA as a substrate of METTL3, and its regulation occurs in an m6A-dependent manner. Additionally, METTL3 was found to modulate the expression of SH3BGR by influencing its mRNA stability. This study unveils a novel mechanism contributing to the dysregulation of genes in DS, particularly shedding light on the upregulation of SH3BGR through post-transcriptional m6A RNA methylation.

The SH3BGR gene is part of a gene family encoding small thioredoxin-like proteins with SH3 domains, predominantly expressed in cardiac and skeletal muscles. Initially identified by Scartezzini et al. [10], SH3BGR was later found to be present during early stages of mouse heart development [18]. Overexpression of Sh3bgr in *Xenopus* embryos resulted in enlarged hearts, underscoring its crucial role in heart development [11]. Notably, SH3BGR is significantly upregulated in the hearts of human patients with cardiac hypertrophy and in a mouse model of heart failure induced by transverse aortic constriction, suggesting its involvement in cardiac hypertrophy [12]. In neonatal rat ventricular cardiomyocytes, overexpression of SH3BGR increases hypertrophic markers (Nppa and Nppb) and cell surface area [12]. However, transgenic mice with an FVB background overexpressing SH3BGR do not exhibit changes in cardiac morphogenesis [20], likely because the transgene only increases gene copy number. In contrast, Down syndrome features abnormal METTL3 expression, which post-transcriptionally enhances SH3BGR mRNA. This results in a more than 7-fold increase in protein levels despite only a 1.5-fold increase in mRNA, leading to a non-gene dosage effect that contributes to abnormal heart development in DS.

In the present and another study [21], SH3BGR was significantly upregulated in fetal heart tissue and DS cardiomyocytes, respectively, suggesting a potential role of SH3BGR in DS-CHD. DS-CHD is a complex condition. Although the ratio of atrioventricular septal defects is striking in DS-CHD, the spectrum of heart defects is varied [22]. In this study, we observed a thin ventricular wall in the majority, and we also showed some ventricular septal defects and an enlarged heart in cKO mice. These results suggested that the increased expression of SH3BGR was highly associated with DS cardiac development.

Heart development is a complex process involving programmed cell death, or apoptosis, which plays pivotal roles in both heart development and disease [23, 24]. Excessive cellular apoptosis can lead to pathological conditions. Previous studies have associated SH3BGR with apoptosis in neonatal rat cardiomyocytes [12]. Considering the heightened oxidative stress levels observed in DS pathogenesis [25], leading to cellular apoptosis, our study observed increased apoptosis in E18.5 cKO mouse cardiac tissue. Notably, this apoptotic increase was mitigated in AC16 cells with a double knockdown of METTL3 and SH3BGR. These findings strongly suggest that METTL3, in regulating SH3BGR, influences heart development through its impact on apoptosis.

In conclusion, we identified METTL3 as a regulator of SH3BGR in DS cardiac tissues in an m6A-dependent manner by affecting its mRNA stability. This enriches our understanding of the mechanisms behind the increased expression of SH3BGR in Down syndrome, suggesting the m6A modification of SH3BGR could serve as a potential therapeutic target. The involvement of METTL3 in regulating SH3BGR is intricately linked to apoptosis in DS heart development, underscoring the need for a deeper understanding of developmental mechanisms for improved therapeutic strategies.

## Materials and methods

### Tissue collection

The research conducted in this study was approved by the Research Ethics Committee of the Henan Provincial Peoples’ Hospital (record number: 2019134). Informed consent for fetal tissue collection and research was obtained from all participants. The DS fetuses were diagnosed by amniocentesis, while diploid fetuses with no genetic abnormality identified by prenatal testing worked as controls. Four fetuses of 18–25 gestational weeks diagnosed as DS or three fetuses diagnosed as diploid via karyotype analysis were collected, respectively. Apical part of the free left ventrical wall (LV) was dissected from these fetuses.

### RNA m6A quantification

Total RNA was extracted from cardiac tissue using Trizol (Invitrogen). The global m6A content in the total RNA was examined by the m6A RNA Methylation Quantification Kit (Epigentek) according to manufacturer’s instruction. The absorbance at 450 nm was measured on a microplate reader. The formula of m6A% = [(sample OD – NC OD) ÷ S]/[(PC OD – NC OD) ÷ P] × 100% was used to calculated the percentage of m6A in total RNA, where S is the amount of sample RNA in nanograms, and P is the amount of positive control in nanograms.

### RNA-Seq and data analysis

Total RNA was extracted via Trizol (Invitrogen). Poly(A) RNA was purified via GenElute mRNA Miniprep kit (Sigma). Library construction was generated by TruSeq Stranded mRNA Sample Prep Kit (Illumina) and then sequenced on the Illumina HiSeq system. Raw reads of each sample were trimmed to remove adaptor sequences and low-quality bases. Sequence reads were normalized using Cufflinks. Differentially expressed genes (DEGs) were identified by cuffdiff with the the thresholds of fold change greater than 2 and adjusted p-value less than 0.05. Gene Ontology (GO) and KEGG analysis was performed via DAVID online. Gene Set Enrichment Analysis (GSEA) analysis was performed via GSEA software (version 4.3.2).

### Reverse transcript quantitative PCR (RT-qPCR)

Total RNA was extracted from fetal cardiac tissue and AC16 cell line. The RNA was reverse transcribed into cDNA via RevertAid First Strand cDNA Synthesis Kit (Thermo Scientific). Quantitative PCR (qPCR) was performed in a Applied Biosystems Stepone Real-Time PCR System with SYBR Green Master Mix (Applied Biosystems). Primers are as follows: METTL3 forward: 5′-CAAGCTGCACTTCAGACGAA-3′; METTL3 reverse: 5′- GCTTGGCGTGTGGTCTTT-3′; GAPDH forward: 5′-GTCTCCTCTGACTTCAACAGCG-3′; GAPDH reverse: 5′-ACCACC CTGTTGCTGTAGCCAA-3′; SH3BGR forward: 5′- GGAAGAAATAGCCATGGAGGGT-3′; SH3BGR reverse: 5′-CCAGGTCAGGCAGCATAAGT-3′.

### MeRIP-quantitative PCR (MeRIP-qPCR)

MeRIP-qPCR was performed as described previously [9]. In brief, the RNA was incubated with anti-m6A antibody. The immunoprecipitated RNA was reverse transcribed and qPCR was performed with specific primers to quantify the enrichment of m6A-containing RNA.

### Western blotting

Western Blotting was conduced as described earlier [9]. The primary antibodies used in this study were diluted at 1:1000 for anti-METTL3 antibody (Abcam, MA5-27527), anti-FTO antibody (Abcam, MA5-33105), an anti-SH3BGR antibody (Abcam, PA5-99717), an anti-METTL14 antibody (Abcam, MA5-47189), anti-CASPASE 3 antibody (Abcam, PA5-77887), and 1:5000 for anti-GAPDH (Abcam, MA5-35235) or anti-β-ACTIN antibody (Abcam, MA5-44307). HRP-conjugated secondary antibody (Abcam, 31460 and 31430) was diluted at 1:10,000.

### Cell culture, siRNAs, and transfection

The human cadiac cell line AC16 was acquired from the Cell Bank of the Chinese Academy of Sciences (Shanghai, China). The cell was incubated in DMEM:F12 medium supplemented with 10% fetal bovine serum and 1% PS (penicillin and streptomycin) at 37^◦^C under 5% CO2. AC16 cells were authenticated via short tandem repeat profiling and were tested for Mycoplasma every 6 months. METTL3 siRNA was described previously [9]. For knockdown of METTL3, the AC16 cells were transfected with METTL3 siRNAs or negative control siRNA at a final concentration of 50 nM via JetPRIME Transfection Reagent (Polyplus) according to the manufacturer’s instructions.

### Virus production and transduction

For overexpression of METTL3, pCDH lentiviral vectors together with delta 8.9 and VSVG were transfected into 293 T cells. Viruses were collected at 48 hours and 72 hours after transfection and used to infect AC16 cells with Polybrene (8 μg/ml, Sigma). Puromycin was added to the culture medium to select the infected cells after 48 hours.

### Dual-luciferase reporter and mutagenesis

Wild-type SH3BGR 3′ UTR or mutant SH3BGR 3′ UTR was inserted into upstream of psiCHECK2 dual-luciferase vector. For dual-luciferase reporter assay, cells were cotransfected with wild-type or mutant SH3BGR 3′ UTR and METTL3 or control siRNA, respectively. The activities of firefly luciferase and renilla luciferase were tested via Dual-Luciferase Reporter Assay System (Promega) 24 h after transfection according to the manufacturer’s protocol.

### Measurement of mRNA stability

AC16 cells were transfected with METTL3 or control siRNAs incubated with 5 μg/ml actinomycin D for 0, 3, 6, 8, and 10 h. RNA was extracted from the cells. Analysis of the half-life of target mRNA was conducted via RT-qPCR as described previously [9].

### Animals, genotyping and embryonic heart dissection

All the animals were housed in a pathogen-free environment. All mice used in this study were on the C57BL/6 N genetic background. All the animal procedures were carried out according to protocols approved by the Zhengzhou University Institutional Animal Care and Use Committee of and the Research Ethics Committee of Zhengzhou University (Zhengzhou, Henan, China). For genotyping, genomic DNA was extracted via the Mouse Genotyping Kit (Vazyme). Briefly, mouse tissues were mixed with 100 μl Buffer and 2 μl Protease, then incubated at 55 °C for 30 min and 100 °C for 10 min. The solution is used for PCR templates. To identify the insertion of loxP sites in *Mettl3*, the PCR products were 290 bp/234 bp (loxP/wt) in *Mettl3* intron 1 with primers F1/R1 and 434 bp/327 bp (loxP/wt) in *Mettl3* intron 3 with primers F2/R2. The loxP inserted both in *Mettl3* intron 1 and intron 3 in the same allele of *Mettl3* were identified as *Mettl3*^*flox/+*^. The loxP inserted both in *Mettl3* intron 1 and intron 3 in both alleles of *Mettl3* were identified as *Mettl3*^*flox/flox*^.

The PCR product for *Myh6-Cre* was 329 bp with primers Myh6-Cre-F/Myh6-Cre-R. F1: 5′-TCCAAGAGTCTAATATCCACCAGCAC-3′; R1: 5′-TGATCAGCAAATGATGGTCCCAG; F2: CCTTCCCCAGATGAAACTGTCTA; R2: GAAAGGCACAGCACTAGTCTTC. Myh6-Cre-F: 5′- CCTGCTGTCCATTCCTTATTCCATA -3′; Myh6-Cre-R: 5′- ATATCCCCTTGTTCCCTTTCTGC-3′. After dissection of mouse embryonic heart, histological examination and ventricular wall thickness measurements were carried out double-blinded. Cardiac tissues were dissected at E18.5 to further undergo RNA-seq analysis.

### Apoptosis detection by flow cytometry

For cell apoptosis, AC16 cells were seeded in 24-well plate and were transfected with siRNAs. 24 h after transfection, cells were measured the cellular apoptosis according to the manufacturer’s instruction. Briefly, the cells were resuspended in annexin binding buffer, then were added with annexin V and propidium iodide (PI) to incubate at room temperature for 15 min in dark. The numbers of apoptotic cells were quantified using Annexin V-FITC Apoptosis Detection Kit (Beyotime) by a flow cytometer (Beckman CytoFLEX). The data were further analyzed by FLOWJO 10.4 (BD Biosciences).

### Statistical analysis

In each set of experiments, the sample size was chosen to ensure adequate power of detection. The variance was similar between the groups that are being statistically compared. The results were expressed as mean ± SD. Student t-test or two-way analysis of variance were used to analyze the statistical significance via GraphPad Prism 8 (GraphPad Software). The level of statistically significance was defined as *p* < 0.05. (*P < 0.05; *P < 0.01; ***P < 0.001)

## Supplementary information

Mettl3 is required for cardiac development

Heatmap of overlapping genes related to heart development or inflammatory response

Legend of supplementary

The list of genes with differential expression located on chromosome 21

The list of genes with differential expression in METTL3-depleted AC16 cells

Original western blots

## Data Availability

RNA sequencing data of Mettl3 cKO mice and METTL3 deficiency AC16 cells have been deposited on SRA of NCBI (PRJNA1136072 and PRJNA1137229, respectively). RNA-sequencing data of human fetal cardiac tissue have been deposited on Genome Sequence Archive of China National Center for Bioinformation with identifier HRA008031.
