# Supplementary figures and images for: Methyltransferase METTL3 governs the modulation of SH3BGR expression through m6A methylation modification, imparting influence on apoptosis in the context of Down syndrome-associated cardiac development
Source: Cell Death Discov. 2024 Sep 6;10:396. doi: 10.1038/s41420-024-02164-3 (PMC11377721; doi:10.1038/s41420-024-02164-3)

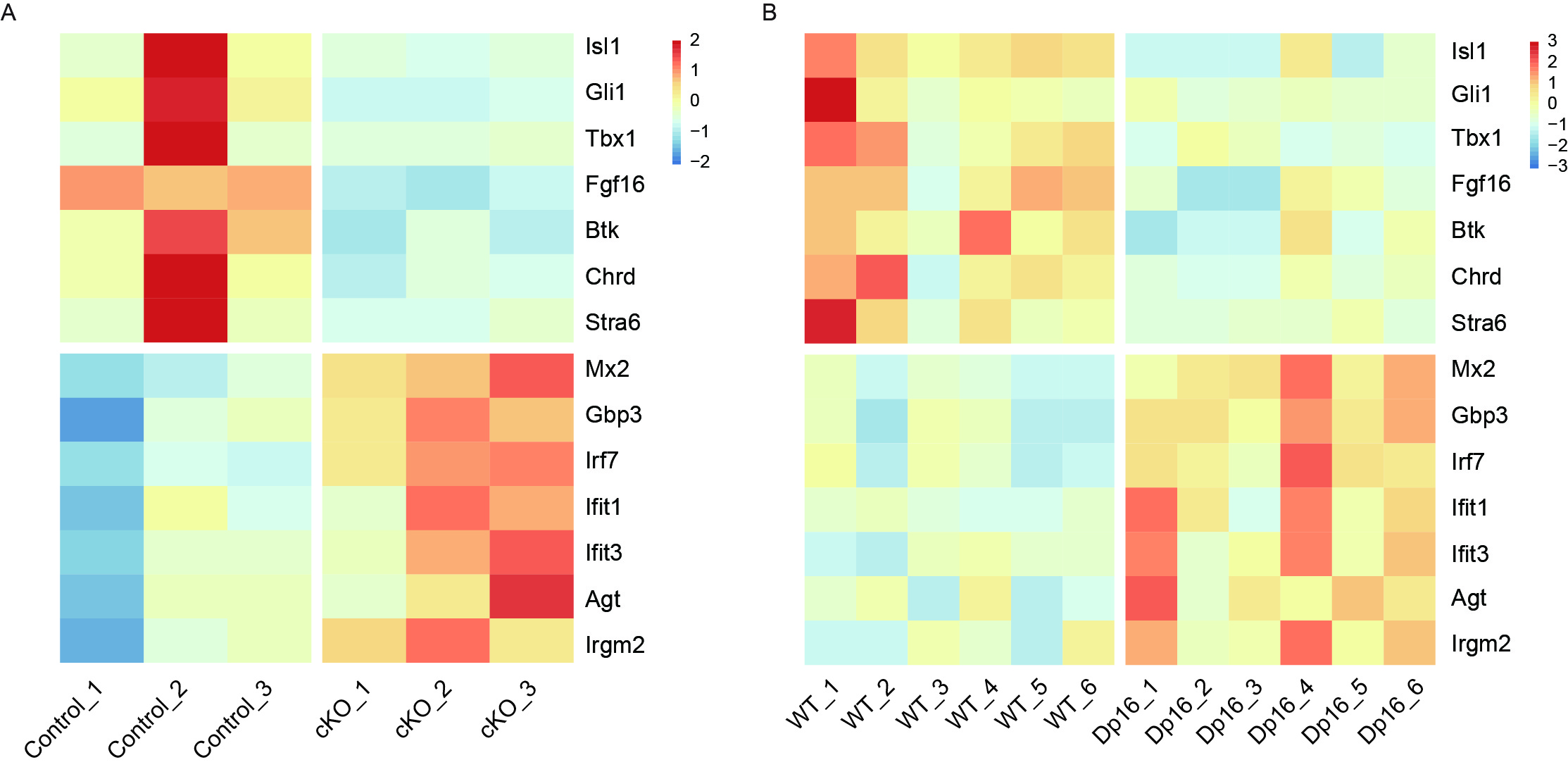

Supplement: Supplementary file 1 — Mettl3 is required for cardiac development [file 41420_2024_2164_MOESM1_ESM.jpg]

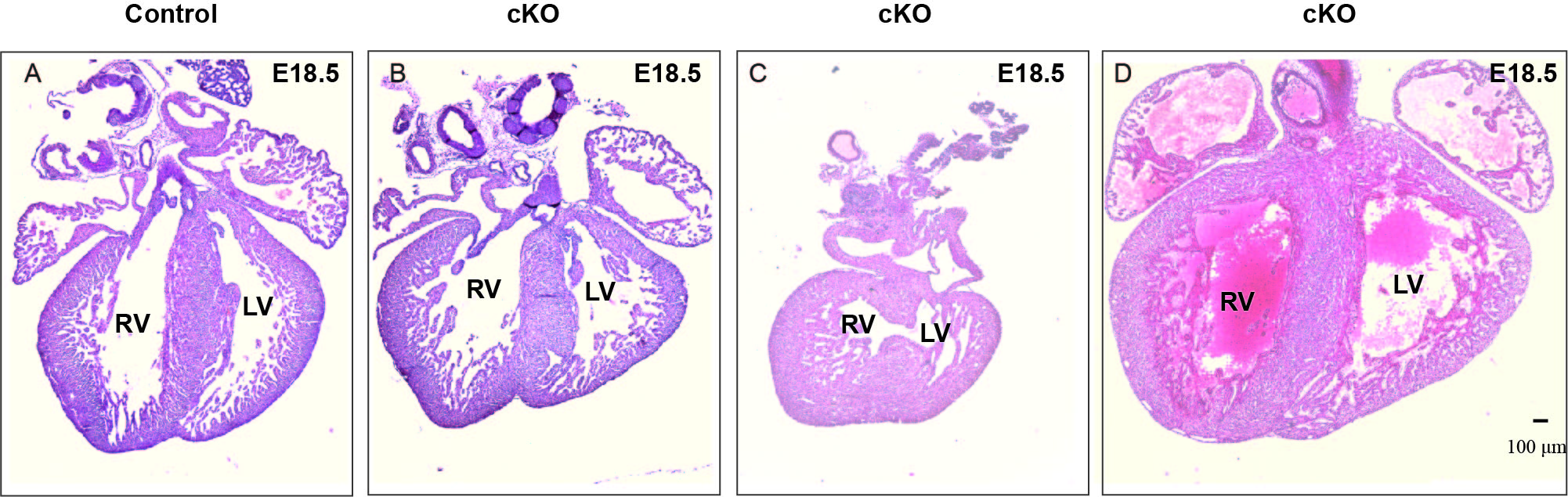

Supplement: Supplementary file 2 — Heatmap of overlapping genes related to heart development or inflammatory response [file 41420_2024_2164_MOESM2_ESM.jpg]

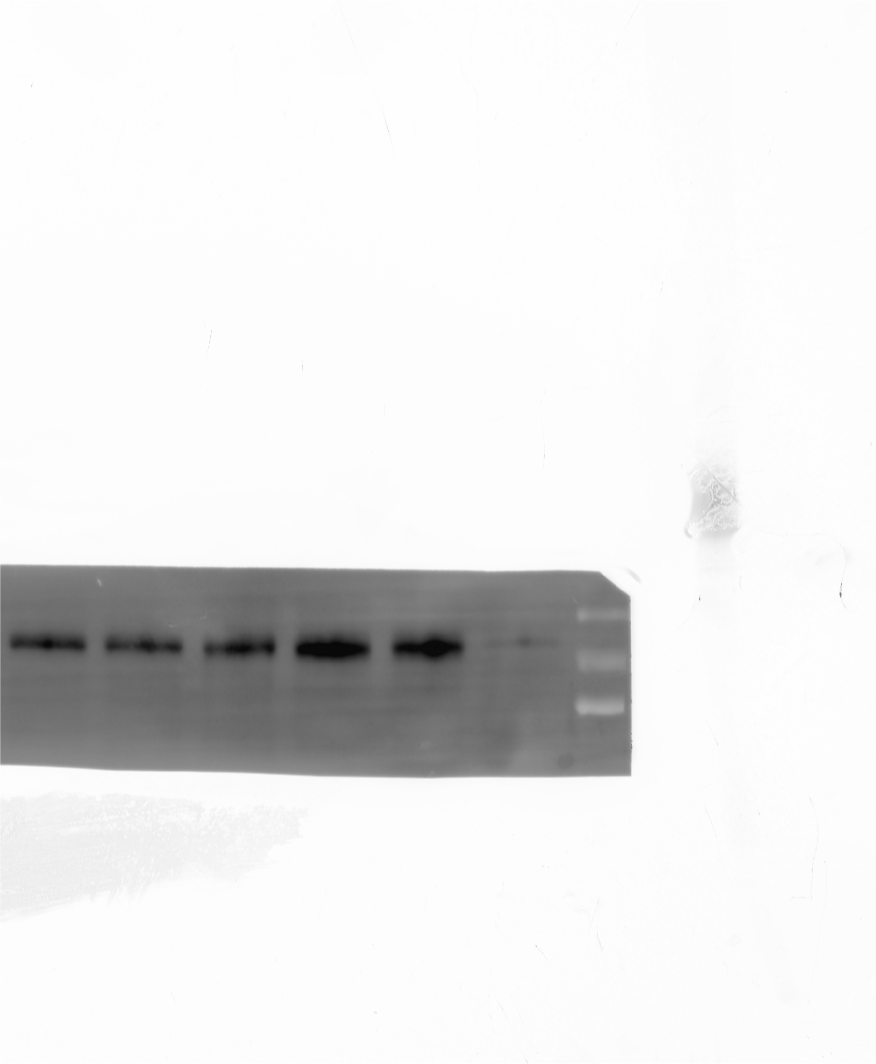

Supplement: Supplementary file 6 — Original western blots [file 41420_2024_2164_MOESM6_ESM.zip › original WB 0424/(Fig 4 B) Mettl3 in cko mice.tif]

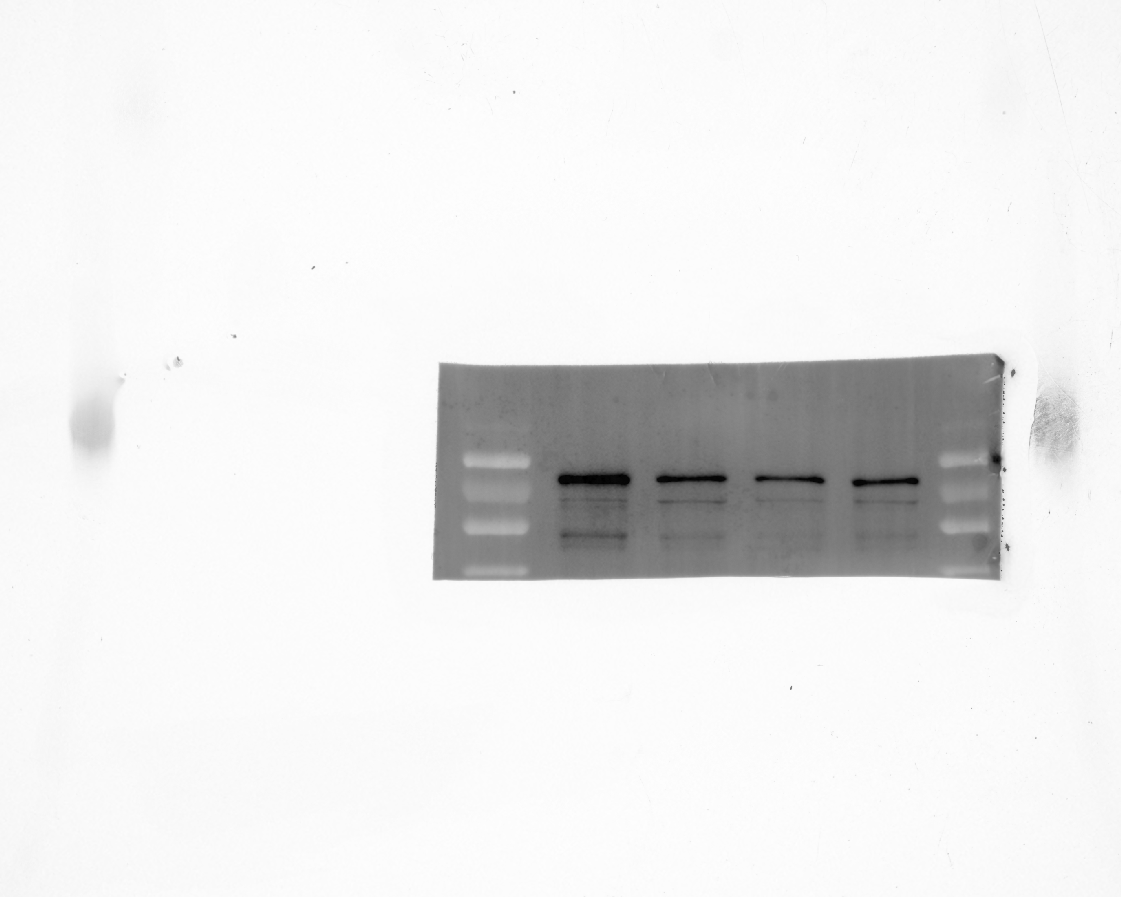

Supplement: Supplementary file 6 — Original western blots [file 41420_2024_2164_MOESM6_ESM.zip › original WB 0424/(Fig 5 D) METTL3 in METTL3 depletion AC16 cells.tif]

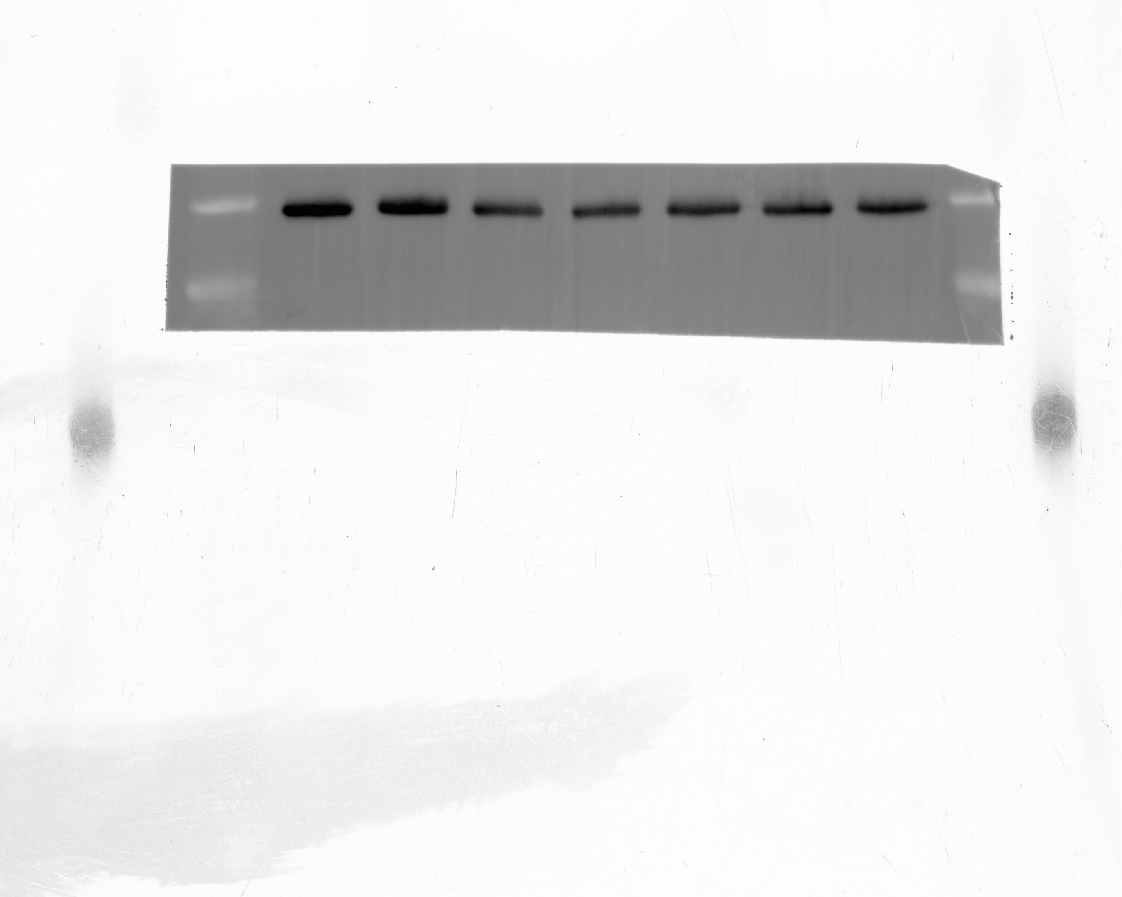

Supplement: Supplementary file 6 — Original western blots [file 41420_2024_2164_MOESM6_ESM.zip › original WB 0424/(Fig 1 B) GAPDH in heart ds.tif]

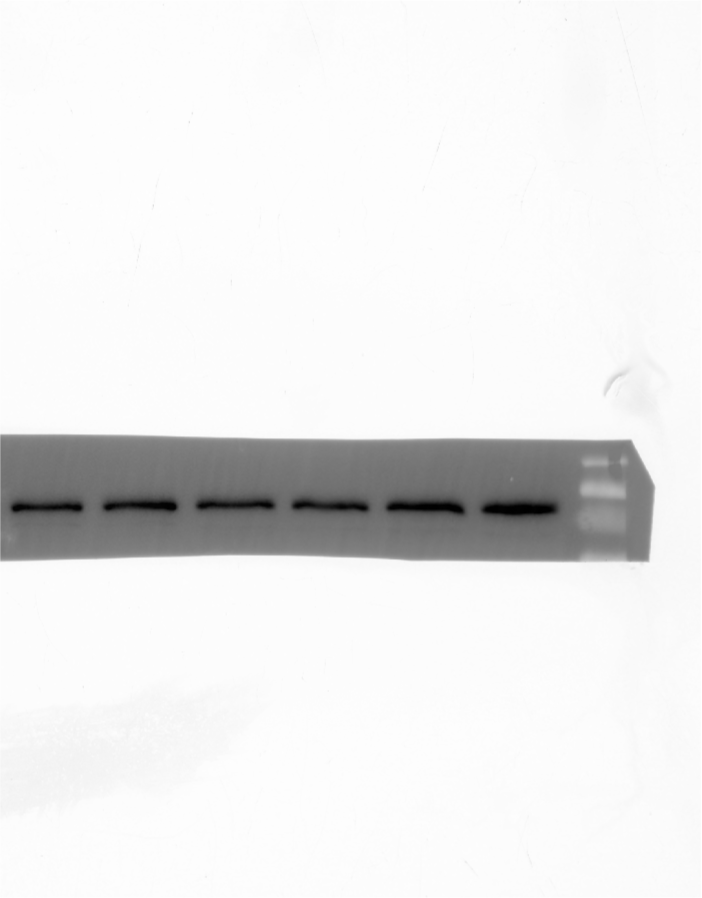

Supplement: Supplementary file 6 — Original western blots [file 41420_2024_2164_MOESM6_ESM.zip › original WB 0424/(Fig 3 C) GAPDH in DS heart.tif]

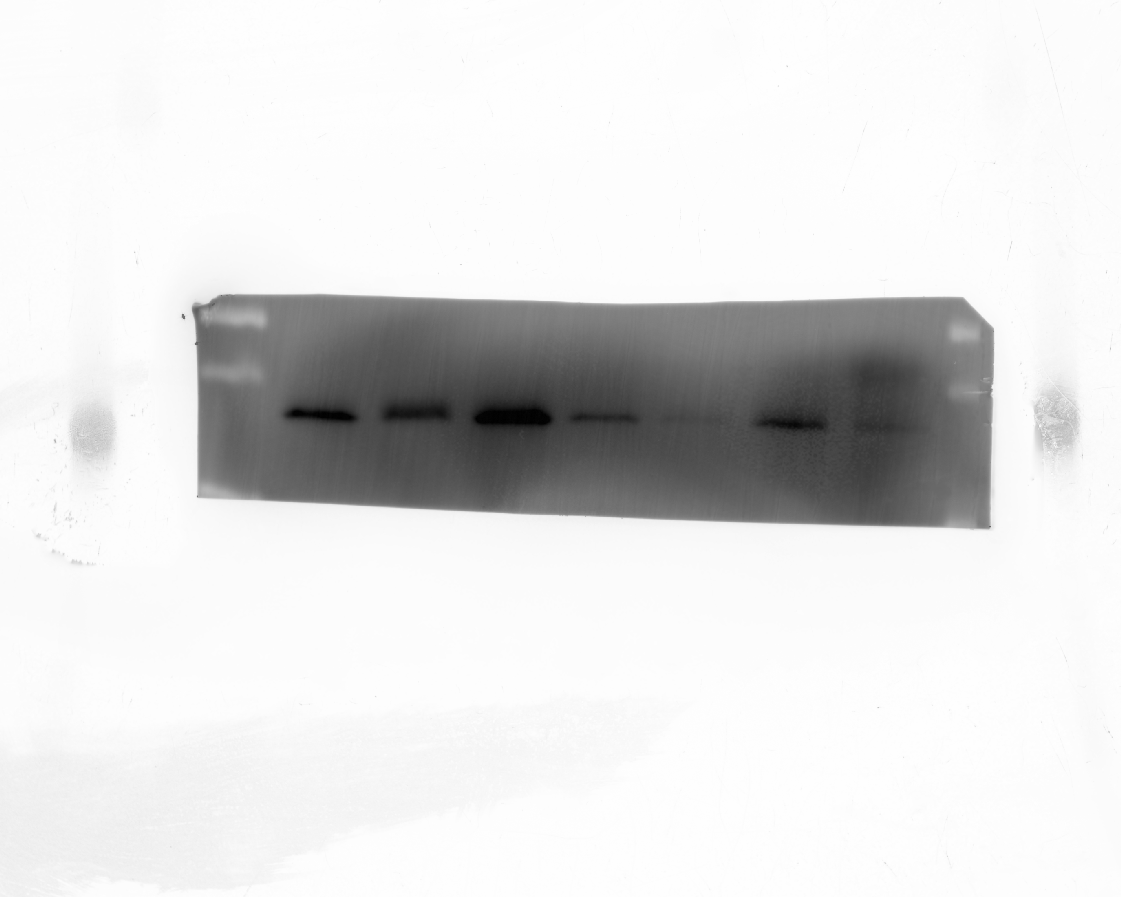

Supplement: Supplementary file 6 — Original western blots [file 41420_2024_2164_MOESM6_ESM.zip › original WB 0424/(Fig 1 B) YTHDF3 in heart ds.tif]

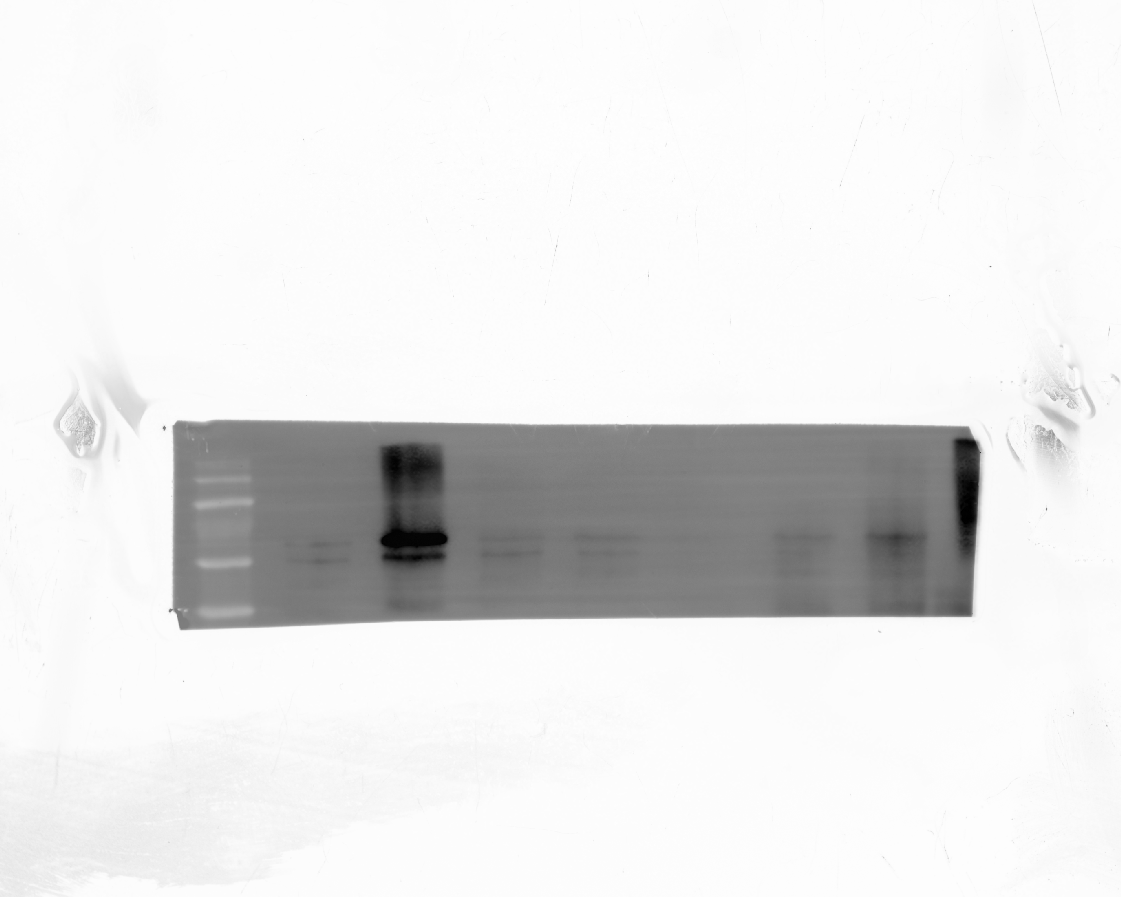

Supplement: Supplementary file 6 — Original western blots [file 41420_2024_2164_MOESM6_ESM.zip › original WB 0424/(Fig 1 B) METTL14 in heart ds.tif]

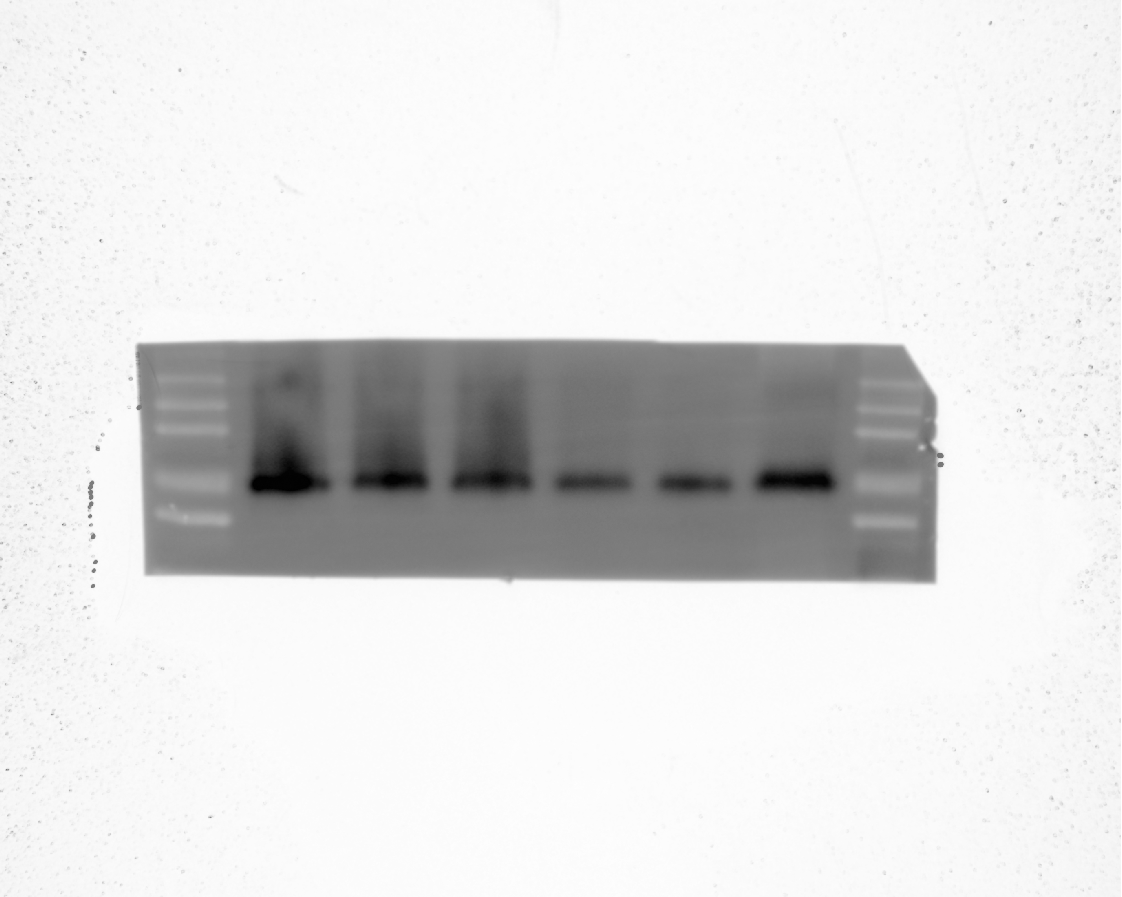

Supplement: Supplementary file 6 — Original western blots [file 41420_2024_2164_MOESM6_ESM.zip › original WB 0424/(Fig 8 F) Sh3bgr.tif]

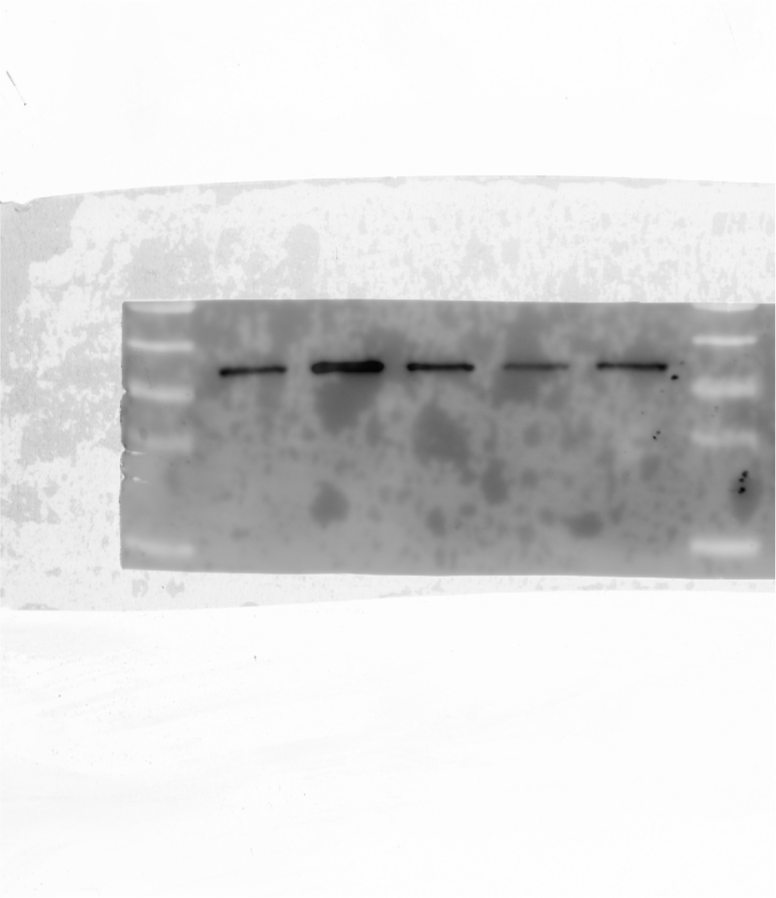

Supplement: Supplementary file 6 — Original western blots [file 41420_2024_2164_MOESM6_ESM.zip › original WB 0424/(Fig 7 C) SH3BGR in AC16 cells relating to apoptosis.tif]

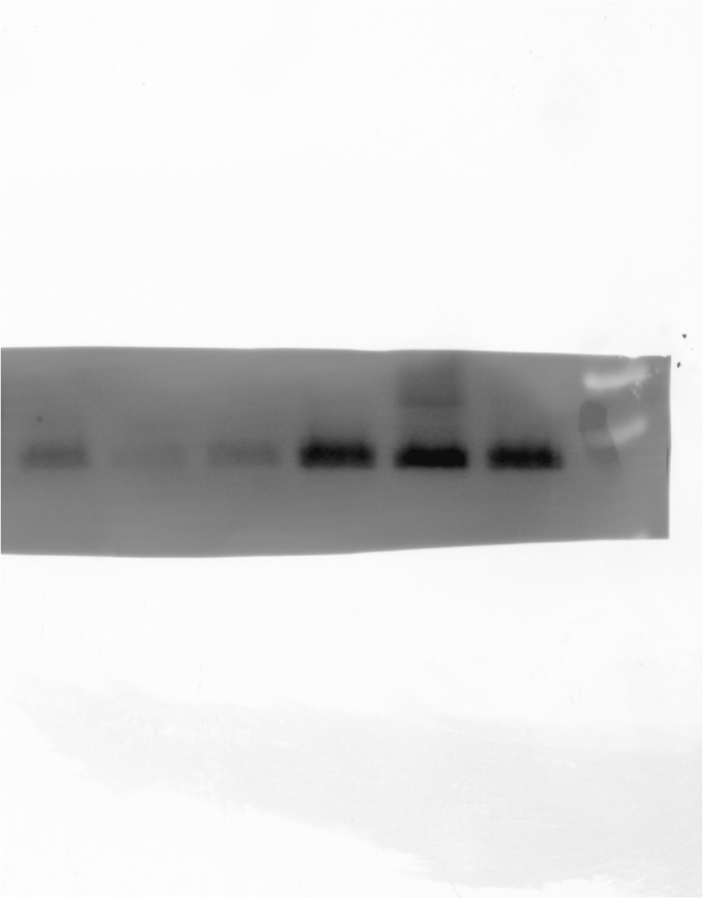

Supplement: Supplementary file 6 — Original western blots [file 41420_2024_2164_MOESM6_ESM.zip › original WB 0424/(Fig 3 C) SH3BGR in DS heart.tif]

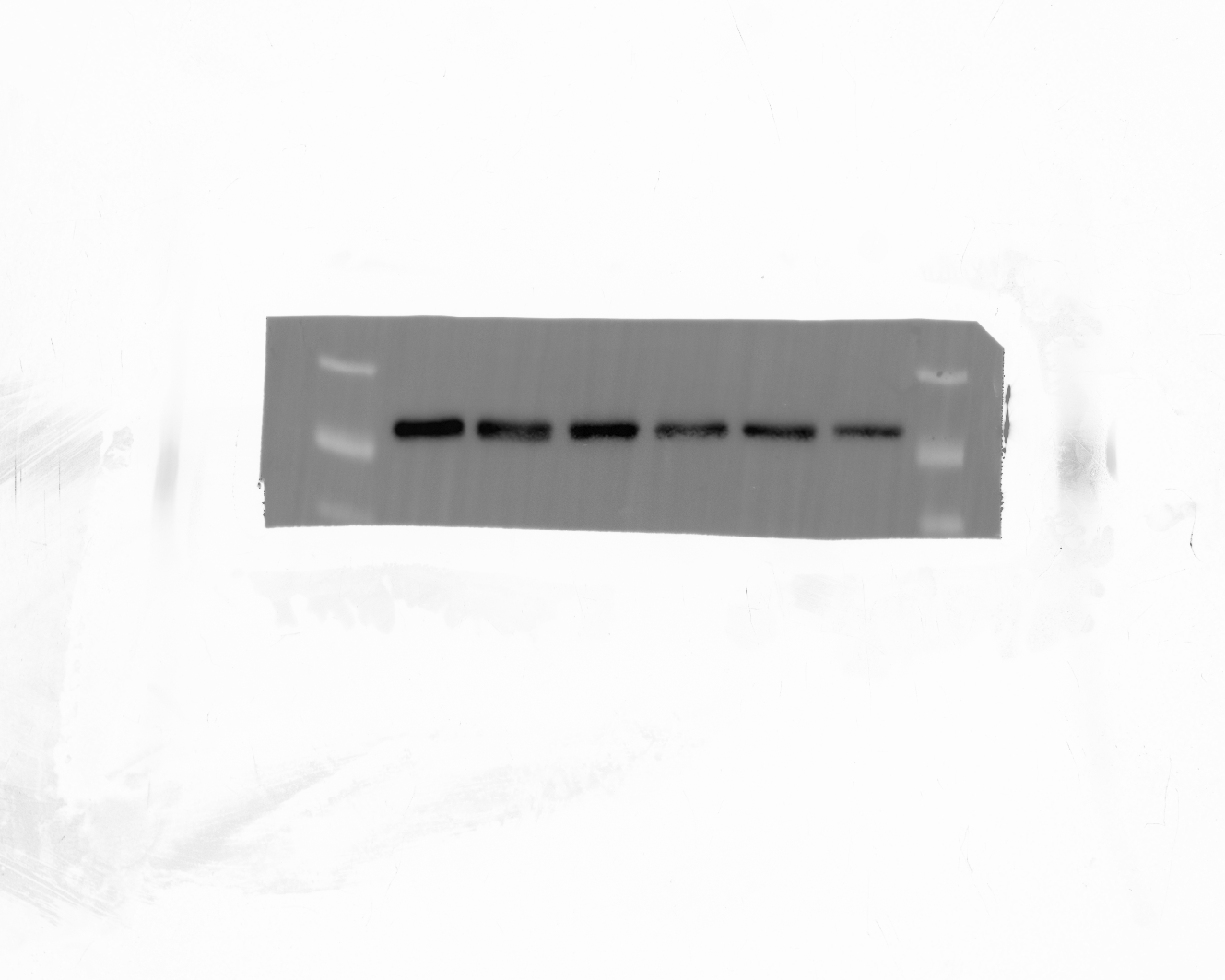

Supplement: Supplementary file 6 — Original western blots [file 41420_2024_2164_MOESM6_ESM.zip › original WB 0424/(Fig 8 A) Actin in cko mice.tif]

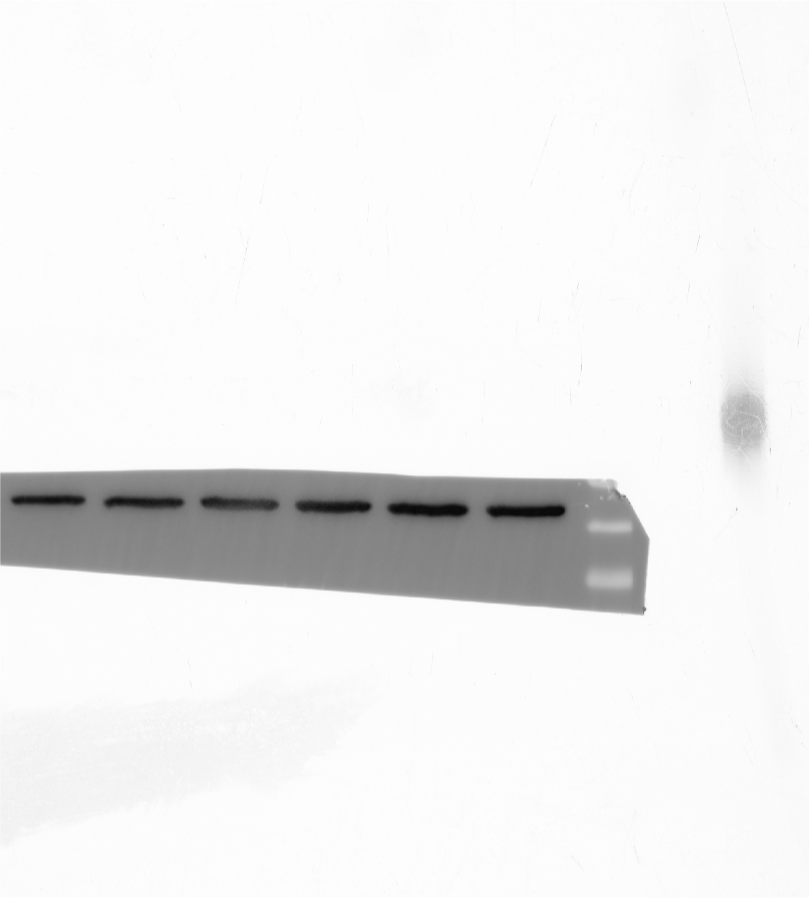

Supplement: Supplementary file 6 — Original western blots [file 41420_2024_2164_MOESM6_ESM.zip › original WB 0424/(Fig 4 B) Gapdh in cKO mice.tif]

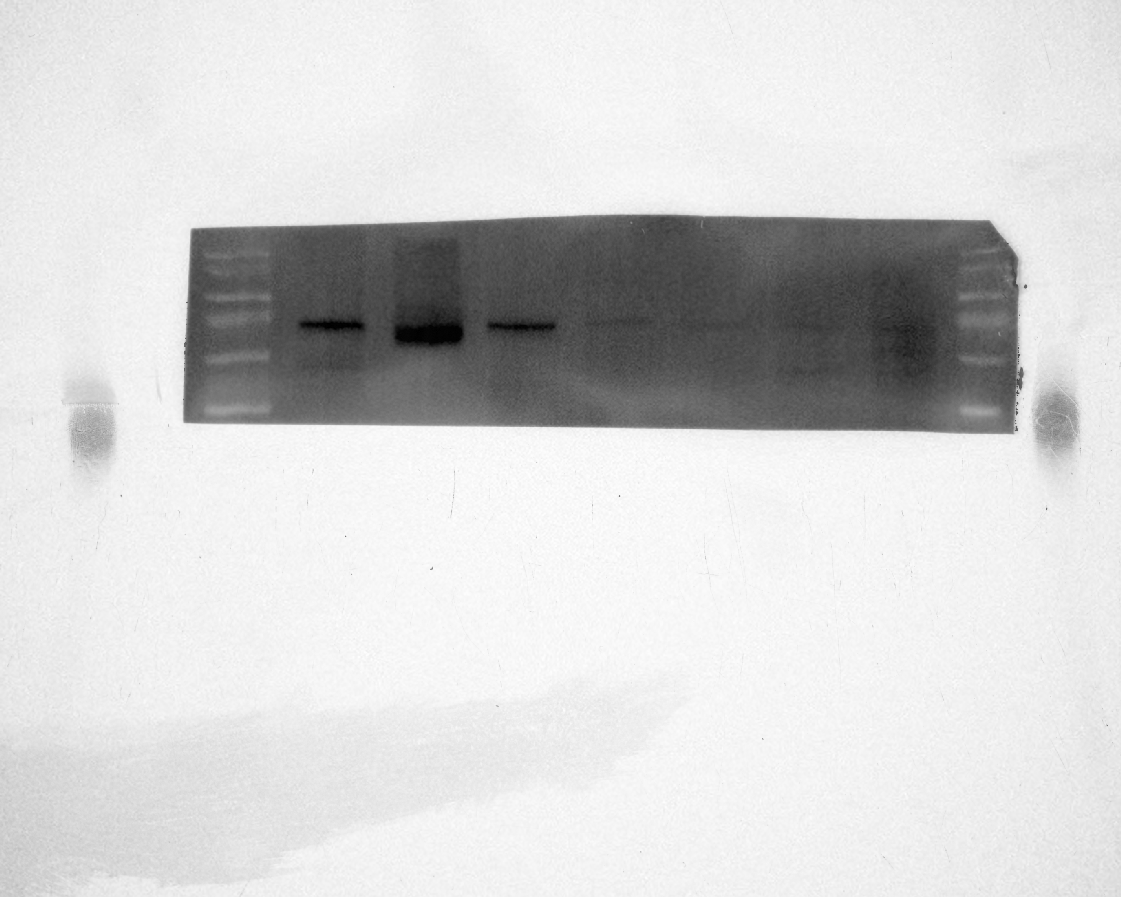

Supplement: Supplementary file 6 — Original western blots [file 41420_2024_2164_MOESM6_ESM.zip › original WB 0424/(Fig 1 B) METTL3 in heart ds.tif]

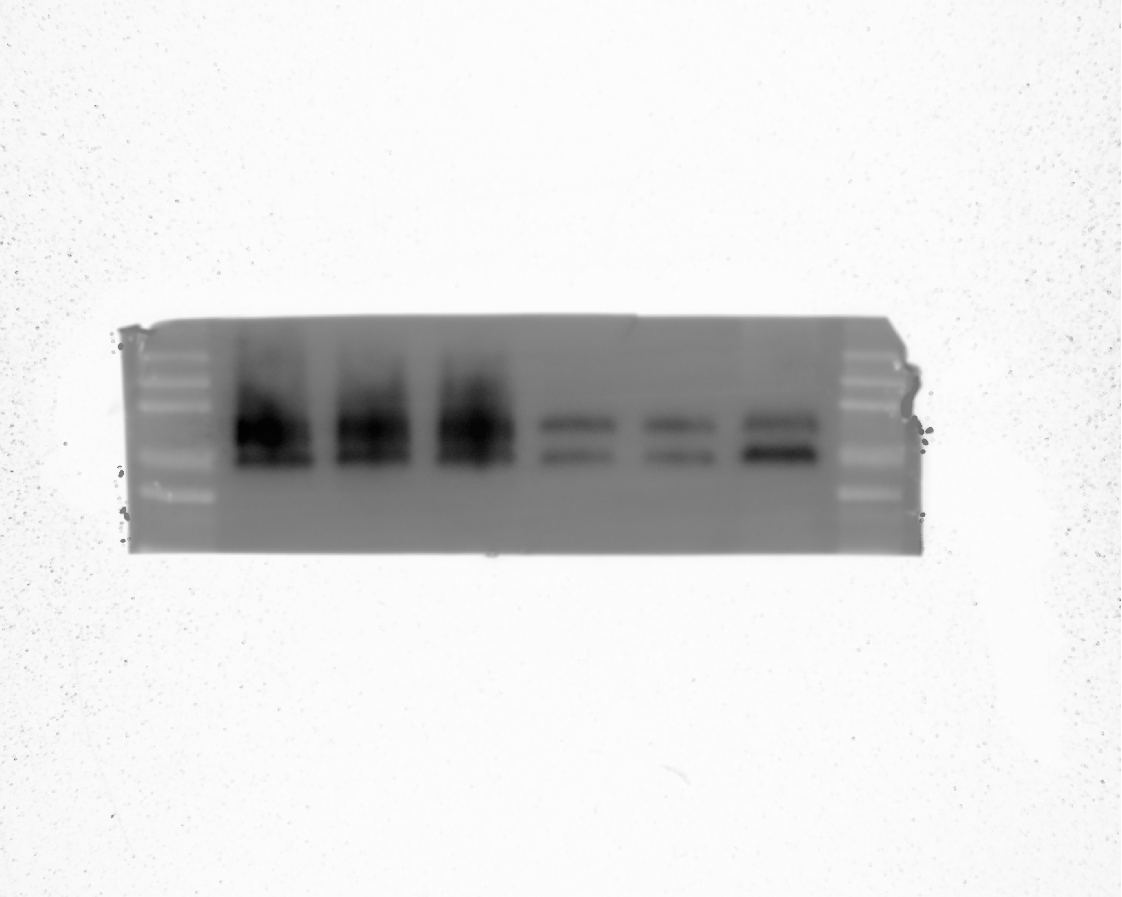

Supplement: Supplementary file 6 — Original western blots [file 41420_2024_2164_MOESM6_ESM.zip › original WB 0424/(Fig 8 F) Cas3.tif]

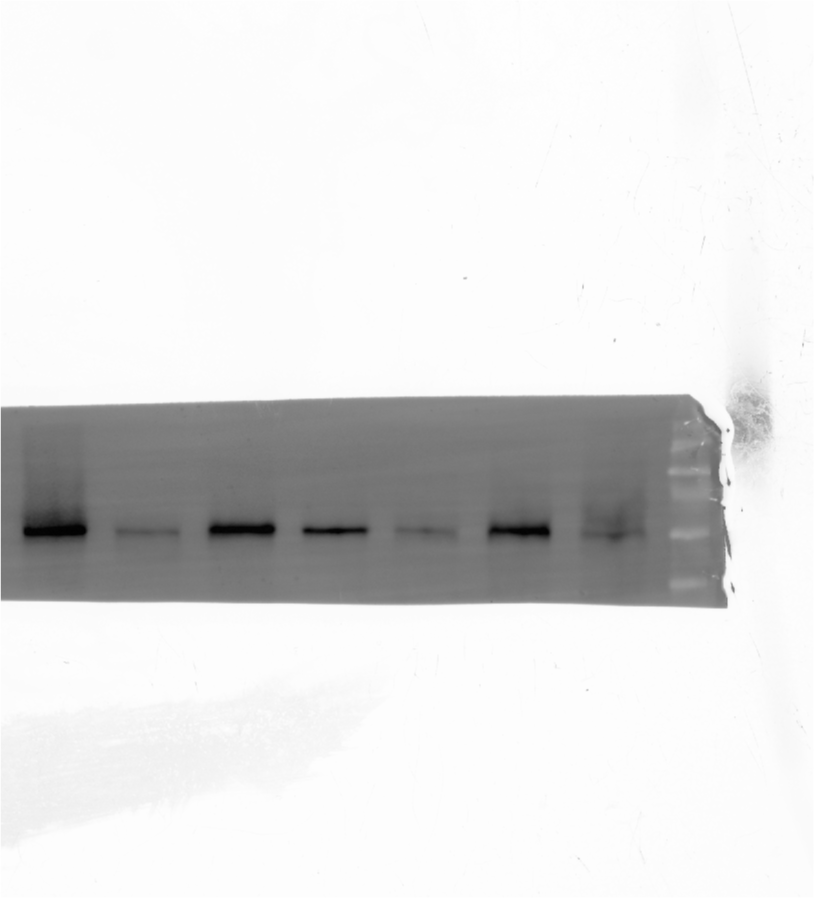

Supplement: Supplementary file 6 — Original western blots [file 41420_2024_2164_MOESM6_ESM.zip › original WB 0424/(Fig 1 B) FTO in heart ds.tif]

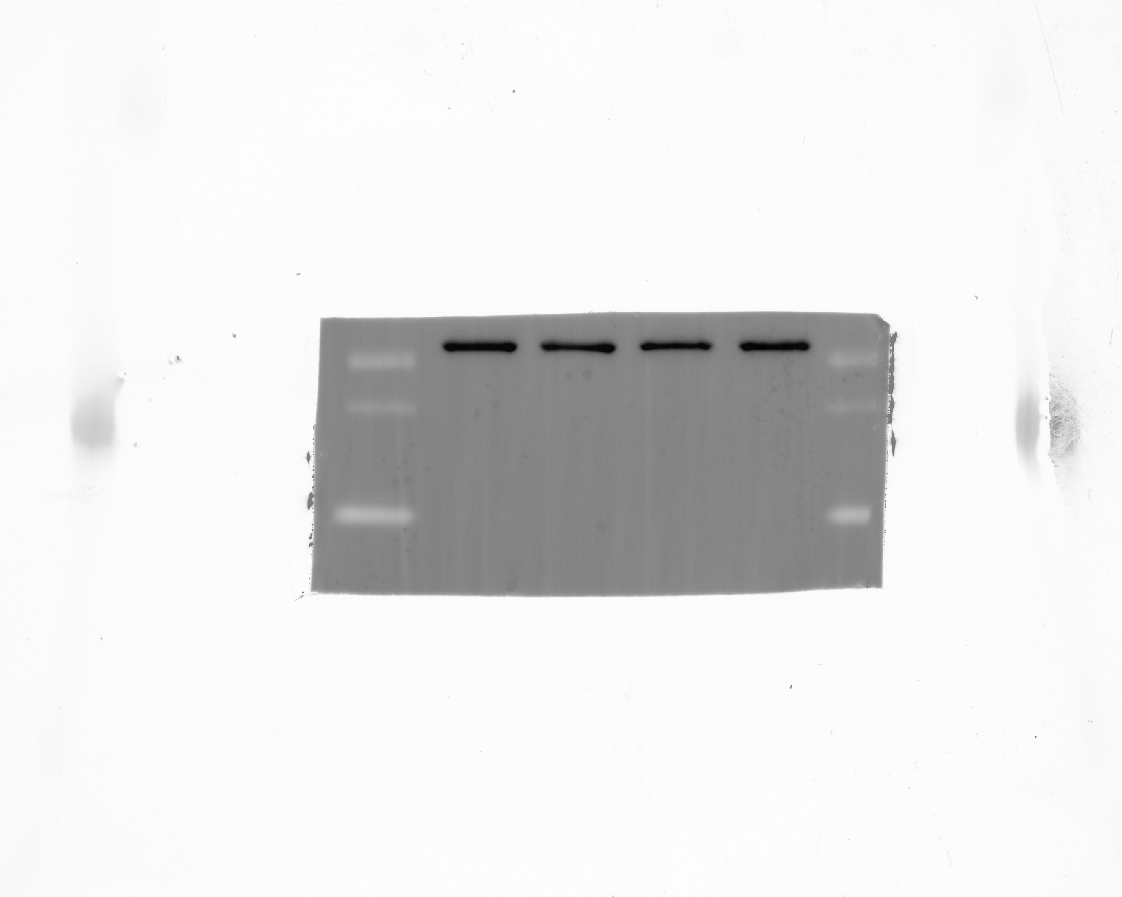

Supplement: Supplementary file 6 — Original western blots [file 41420_2024_2164_MOESM6_ESM.zip › original WB 0424/(Fig 5 D) ACTIN in METTL3 depletion AC16 cells.tif]

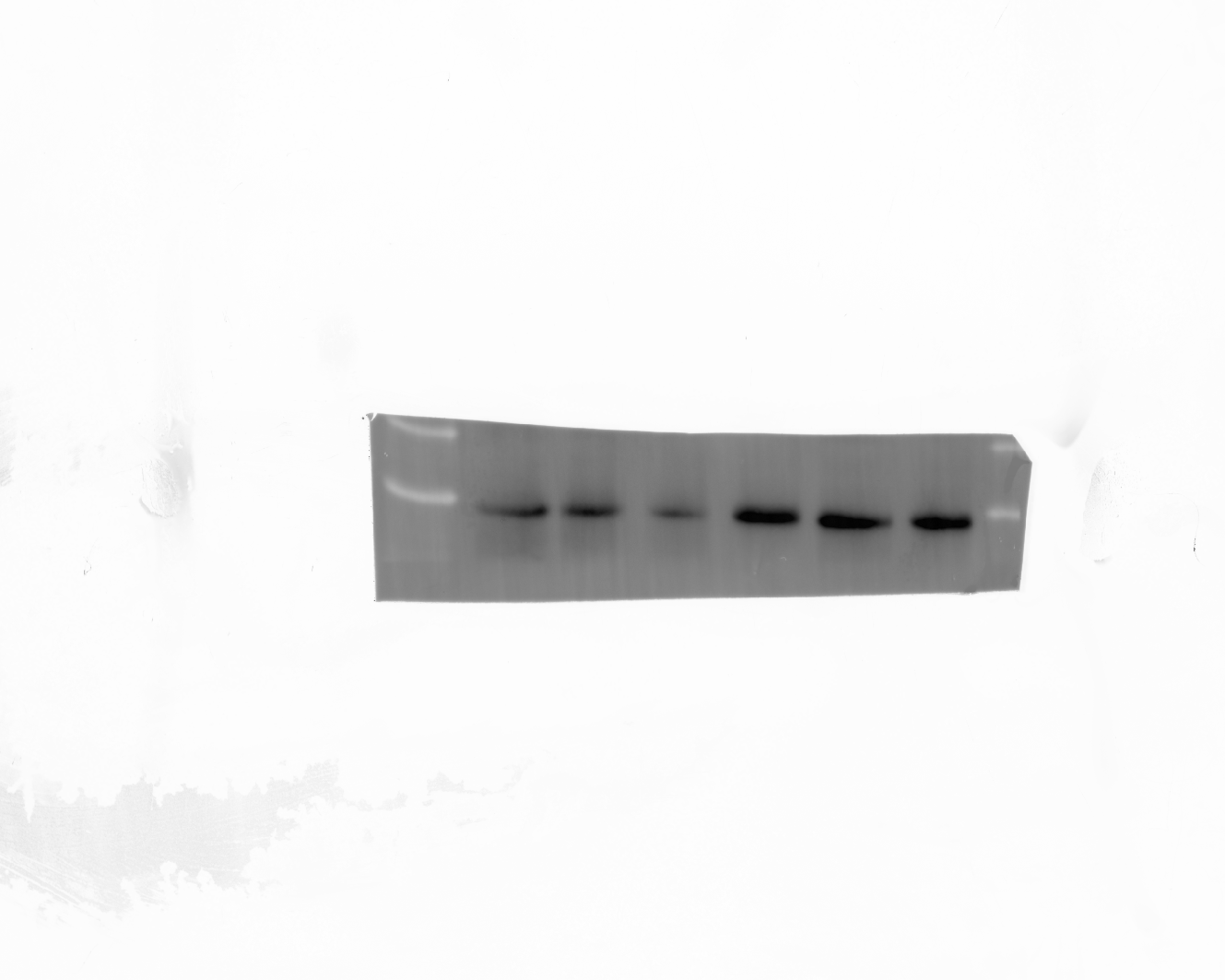

Supplement: Supplementary file 6 — Original western blots [file 41420_2024_2164_MOESM6_ESM.zip › original WB 0424/(Fig 8 A) Sh3bgr in cko mice.tif]

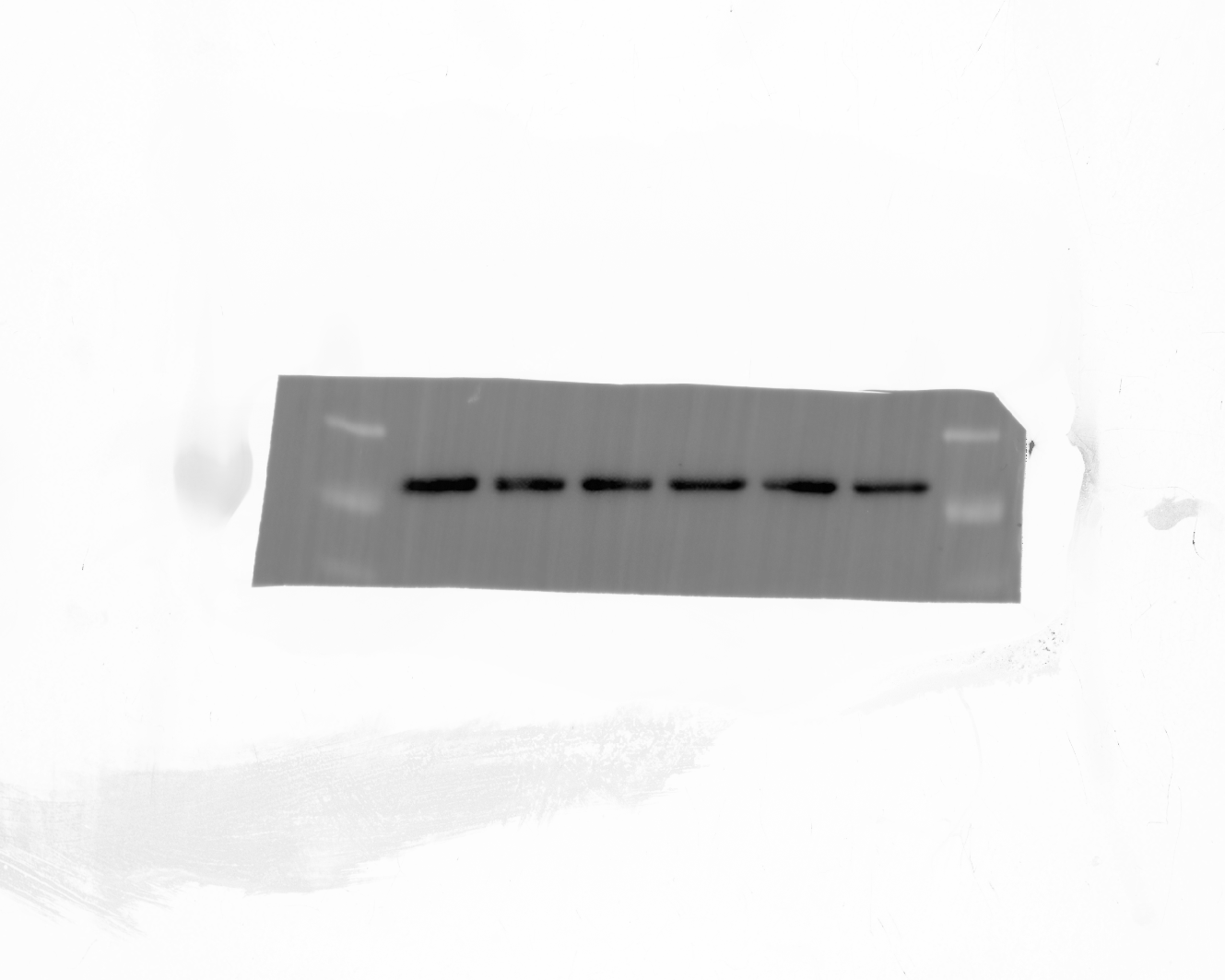

Supplement: Supplementary file 6 — Original western blots [file 41420_2024_2164_MOESM6_ESM.zip › original WB 0424/(Fig 8 F) actin.tif]

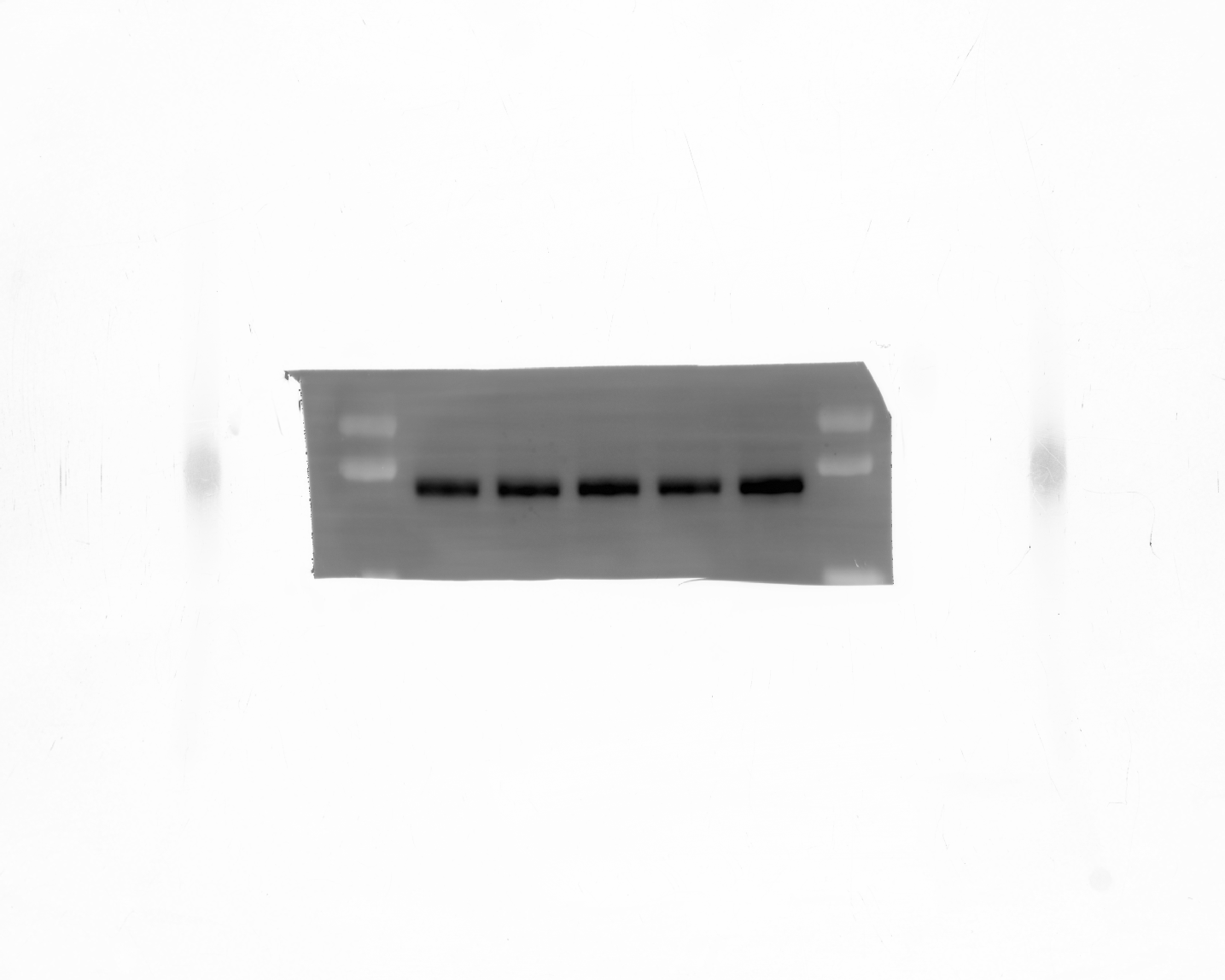

Supplement: Supplementary file 6 — Original western blots [file 41420_2024_2164_MOESM6_ESM.zip › original WB 0424/(Fig 7 C) GAPDH in AC16 cells relating to apoptosis.tif]

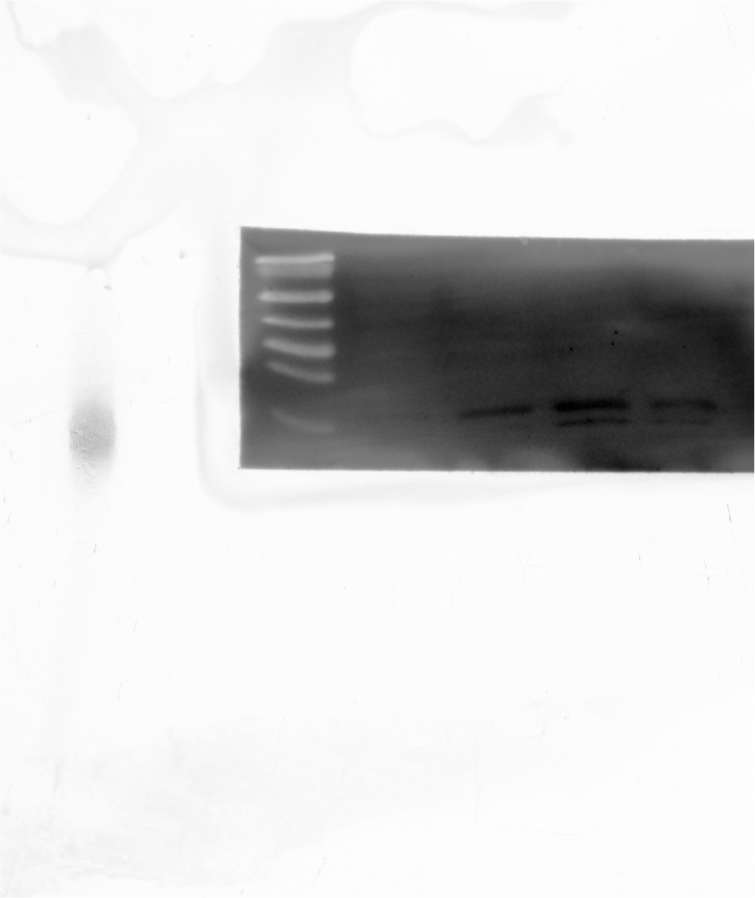

Supplement: Supplementary file 6 — Original western blots [file 41420_2024_2164_MOESM6_ESM.zip › original WB 0424/(Fig 5 D) SH3BGR in METTL3 depletion AC16 cells.tif]
