# Supplementary material for: Methyltransferase METTL3 governs the modulation of SH3BGR expression through m6A methylation modification, imparting influence on apoptosis in the context of Down syndrome-associated cardiac development
Source: Cell Death Discov. 2024 Sep 6;10:396. doi: 10.1038/s41420-024-02164-3 (PMC11377721; doi:10.1038/s41420-024-02164-3)
Supplement: Supplementary file 3 — Legend of supplementary [file 41420_2024_2164_MOESM3_ESM.docx]

**Supplementary Fig. 1 Heatmap of overlapping genes related to heart development or inflammatory response**

Heatmap of several overlapping genes related to heart development or inflammatory response. (A) Heatmap of several overlapping genes in cKO mouse. (B) Heatmap of the same overlapping genes in Dp16 mouse.

**Supplementary Fig. 2 Mettl3 is required for cardiac development**

Histological analysis of control and cKO mouse hearts. Compared to control (A), H&E staining of cKO transverse sections showed thinning of the ventricular walls (B), ventricular septal defect (C), and an enlarged heart (D).
